# Modeling causes of death: an integrated approach using CODEm

**DOI:** 10.1186/1478-7954-10-1

**Published:** 2012-01-06

**Authors:** Kyle J Foreman, Rafael Lozano, Alan D Lopez, Christopher JL Murray

**Affiliations:** 1 Institute for Health Metrics and Evaluation, University of Washington, 2301 5th Ave, Seattle, WA 98121, USA; 2 School of Population Health, University of Queensland, Level 2 Public Health, Herston Road, Herston QLD 4006, Australia

**Keywords:** cause of death, ensemble models, predictive validity, spatial-temporal models, maternal mortality, Global Burden of Disease

## Abstract

**Background:**

Data on causes of death by age and sex are a critical input into health decision-making. Priority setting in public health should be informed not only by the current magnitude of health problems but by trends in them. However, cause of death data are often not available or are subject to substantial problems of comparability. We propose five general principles for cause of death model development, validation, and reporting.

**Methods:**

We detail a specific implementation of these principles that is embodied in an analytical tool - the Cause of Death Ensemble model (CODEm) - which explores a large variety of possible models to estimate trends in causes of death. Possible models are identified using a covariate selection algorithm that yields many plausible combinations of covariates, which are then run through four model classes. The model classes include mixed effects linear models and spatial-temporal Gaussian Process Regression models for cause fractions and death rates. All models for each cause of death are then assessed using out-of-sample predictive validity and combined into an ensemble with optimal out-of-sample predictive performance.

**Results:**

Ensemble models for cause of death estimation outperform any single component model in tests of root mean square error, frequency of predicting correct temporal trends, and achieving 95% coverage of the prediction interval. We present detailed results for CODEm applied to maternal mortality and summary results for several other causes of death, including cardiovascular disease and several cancers.

**Conclusions:**

CODEm produces better estimates of cause of death trends than previous methods and is less susceptible to bias in model specification. We demonstrate the utility of CODEm for the estimation of several major causes of death.

## Background

Data on causes of death by age and sex are a critical input into health decision-making. Nations devote considerable resources to collecting, collating, and analyzing various types of cause of death data for this reason [1-3]. Priority setting in public health, however, should be informed not only by the current magnitude of health problems but by trends in them. Whether or not a cause of death is increasing or decreasing is important information as to whether current disease control efforts are working or inadequate. The rising burden of diabetes and the policy debate it has triggered is a good example of the importance of monitoring national trends in causes of death [4,5].

The fundamental challenge for most countries, however, is that cause of death data are often not available or subject to substantial problems of comparability. Even in the 89 countries with complete vital registration systems and medical certification of causes of death in 2009, many issues of comparability remain [6-8]. Dramatic changes from year to year in death rates from a cause can be due to changes in International Classification of Diseases (ICD) revision [9,10] or national modifications of coding rules [11-14]. In some cases, causes such as HIV or diabetes may be systematically misclassified [8,15-21]. The fraction of deaths assigned to causes that are not true underlying causes of death can vary widely and change over time [20,22-27]. In places without complete vital registration, a range of sources such as verbal autopsy studies (national or subnational), partial urban vital registration, or survey/census data may be available. Data may be available only for a limited number of years and these data are often subject to substantial sampling and even larger nonsampling error. Generating national assessments of causes of death by age, sex, and year requires a strategy and methodology to deal with this diverse set of data issues.

Efforts to model causes of death using available data have a long history [28-34]. Initial attempts focused on estimating causes of death for a cross-section of countries by modeling cause as a function of overall mortality levels. For example, Preston's 1976 *Mortality Patterns in National Populations: With Special Reference to Recorded Causes of Death *[28] was the initial effort to assess trends in causes of death taking into account misclassification of deaths. The demand for estimates of both levels and trends in key causes from diverse groups have led to multiple recent studies on diarrhea, maternal mortality, and other causes of death [29-31,35-37]. These studies have used a wide variety of analytical strategies and specific model implementations. The recent debate on maternal mortality estimation [35,36,38-41] is an illustration of quite different choices of the dependent variable and model specifications. The use of Gaussian Process Regression (GPR) and other related techniques has been used for all-cause mortality in children and adults and in time series cross-sectional work on key risk factors [5,42-45]. Affordable computational power and innovations in Bayesian statistical modeling have fueled a steady growth in alternative estimation strategies. This innovation is likely to continue for the foreseeable future.

Comparing alternative modeling approaches applied to the same cause of death is complicated by a lack of accepted standards for good cause of death modeling practice. Preferences for the results of alternative strategies may be based not on documented performance but on impressionistic grounds. In this paper, we propose five general principles for cause of death model development, validation, and reporting. We then detail a specific implementation of these principles that is embodied in an analytical tool, CODEm - the Cause of Death Ensemble model.

### Principles for cause of death model development

#### 1. Identify all the available data

Good cause of death modeling practice begins with a systematic attempt to identify all the available data. Most cause of death data is captured through a variety of national data collection systems such as partial or complete vital registration or national or sample registration systems with verbal autopsy. Most of these data are not published in the scientific literature but are available through national sources or the World Health Organization (in the case of vital registration with medical certification of causes of death). These main sources can also be supplemented with subnational studies on select causes or age groups from the published literature through systematic reviews. For some diseases, there may be special sources of information, such as population-based cancer registry data for mortality from selected cancers in particular catchment areas.

#### 2. Maximize the comparability and quality of the dataset

After all the available data have been identified, several common challenges for the comparability and quality of cause of death data need to be addressed, including mapping across various revisions of the ICD, variation in garbage coding across countries and time, misclassification due to poor diagnostic capacity, comparability of alternative verbal autopsy methods, completeness of cause of death registration, and large nonsampling variance. There is an extensive literature on the mapping for different causes across revisions of the ICD [13,46]; the challenge is greater for certain specific causes of death. A second important source of known bias is the assignment of a substantial fraction of deaths to causes of death that are not underlying causes of death, often called "garbage codes"[27,47-49]. Preston in 1976 already noted that trends in cardiovascular disease over time were profoundly different if garbage codes were taken into account [28,36]. The Global Burden of Diseases, Injuries, and Risk Factors (GBD) 1990 Study introduced simple algorithms for redistributing deaths from major garbage codes [34], and these were refined for the GBD 2000 Study work [50]. More detailed algorithms driven by a more detailed examination of disease pathology have since been proposed [1,51]. Special methods have been proposed for selected causes, such as HIV in populations where the cause is often misclassified due to stigma or other factors. For example, Birnbaum et al. found that many HIV deaths in South Africa had been classified to other causes including tuberculosis, pneumonias, and other infectious diseases [52]. The substantial difference in the strategy for correcting misclassification in two recent studies on maternal mortality illustrates the spectrum of approaches in use [36,39,40]. Uncertainty in the correction for known bias should, in principle, be propagated into the uncertainty in the results. Methods for quantifying this uncertainty, however, have not yet been developed. A third critical factor in enhancing comparability and quality is to correct for the fact that in some vital event registration systems, not all deaths are captured. Death rates based on these systems need to be corrected for the completeness of death registration. Various methods have been proposed to correct for completeness [34,53].

Even after various known biases due to ICD classification changes, garbage coding, and completeness have been taken into account, the results of some studies may be subject to large nonsampling error. Nonsampling error can come about from a wide range of factors that can affect vital registration, verbal autopsy, and other surveillance data sources. Explicit criteria should be used to detect extreme outliers in the rates or cause fractions derived from different sources. Based on explicit criteria, outlier data points should be excluded from the cause of death modeling analysis.

#### 3. Develop a diverse set of plausible models

There are a myriad of choices to be made when modeling cause of death trends. Cause of death data are often sparse for many low-income countries and may also be more available for certain time periods such as 1995 to 2005 than for earlier or later periods; predictions for data-sparse time periods and/or populations are often sensitive to subtle differences in model specification or the form of the dependent variable; modeling cause-specific mortality rates or cause-fractions can yield very different results; and choosing to use educational attainment rather than income per capita can lead to major differences in the predicted trend in causes of death in places with economic downturns. We believe that good modeling practice starts by casting a wide net in terms of proposed models.

Whatever models that are proposed, however, should meet basic plausibility criteria. Known strong biological or behavioral relationships should be respected. For example, models for the age-specific stroke death rate should have a positive coefficient on mean systolic blood pressure of the population, models for lung cancer should have a positive coefficient on tobacco consumption, and so on. A set of models for which these known directions of relationships are respected provides a more robust platform for assessing models and creating model ensembles.

The diverse set of models we test also includes various ways of combining the predictions of multiple individual models. Experience in many fields - ranging from meteorology, soil chemistry, and stocks to the Netflix Challenge and others - have demonstrated that ensemble models have smaller prediction error than the best single model [54-61]. Ensemble models that are weighted combinations of the posterior distributions of component models provide lower error for the point estimate and more accurate uncertainty intervals [62-66]. Additionally, ensemble models capture uncertainty not only due to the parameters in any one model but also the uncertainty of predictions due to differences in specification across models. Many methods have been proposed and used for developing weights for component models in an ensemble, including Bayesian Model Averaging (BMA), averaging of all plausible models, and using fixed or arbitrary weights [67].

#### 4. Assess the predictive validity of each plausible individual model and of ensemble models

In-sample fit is not a robust measure of prediction when data are sparse or missing. Instead, out-of-sample predictive validity is the appropriate measure [66,68-72]. If one model generates better out-of-sample predictions than another, we should prefer this model for the task of prediction. Assessing predictive validity requires holding out some fraction of the data from model building and then assessing the predictions from the model against the data that have been held out. The compelling logic that modeling strategies used for prediction should be assessed through out-of-sample predictive validity is unlikely to be controversial. There are, however, many options for assessing predictive validity that deserve exploration, including which data to hold out, how many repeated samples to perform, and which metrics of validity to calculate.

**Figure 1:**
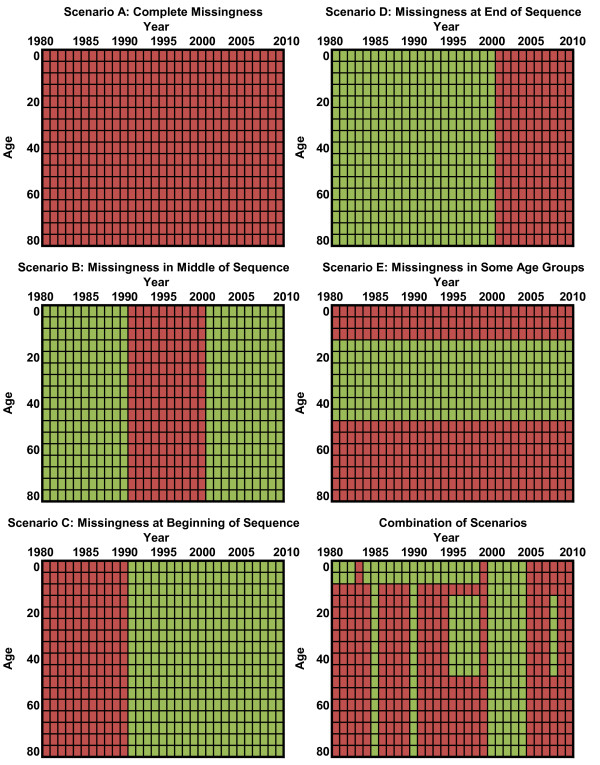
**Examples of missingness patterns observed in data for a single country and cause of death**. Green represents available data and red represents missing.

In bioinformatics and genomics, the standard predictive validity approach is to perform five- or 10-fold cross-validation, in which 10% or 20% of the data are held out and models are developed on the remaining data, a process which is then repeated multiple times to ensure stability. The statistical literature suggests that leave-one-out methods overestimate performance, instead recommending that larger hold-outs be used [73-75]. For cause of death estimation, we can identify five distinct scenarios for prediction: a) countries with no data; b) countries with missing data years but with data from years before and after the missing sequence; c) data missing at the beginning of the sequence; d) data missing at the end of the sequence; and e) data for some age groups such as children or reproductive-age females and not for other age groups. Countries may often be characterized by a combination of these scenarios. Different models can and do in fact perform differentially on these various tasks. We believe that the data hold-out strategy should mimic the task required of the model. In other words, we should hold out sequences of data in proportion to how often the model would need to fill in such a sequence (Figure 1 illustrates the construct). In this way, the hold-out will reflect the appropriate mix of the five scenarios that have been described. Producing train-test datasets through a hold-out strategy should be repeated often enough to yield stable assessments of predictive validity that are not a function of the idiosyncratic nature of a particular randomly generated data hold-out.

The metrics of predictive validity are more complex. Three distinct dimensions should be assessed: errors in predicting levels, errors in predicting trends, and the adequacy of uncertainty intervals. First, for assessing prediction error for the quantity of interest, one can imagine metrics that assess absolute error or relative error and metrics that are based on variances or absolute deviations. Second, two models may yield similar metrics of prediction error on the levels of mortality but one model may more robustly predict the trend in a given cause of death. For many applications of cause of death modeling, estimating an accurate trend may be even more important than estimating an accurate level. Predictive validity for trends can be assessed by examining the first differences over time for the prediction and the data and assessing error in this dimension. Third, it is particularly important to derive uncertainty intervals for cause of death estimation that are accurate. Coverage of the uncertainty intervals of any proposed model should be reported taking into account the sampling and nonsampling error estimated for a model. The reported uncertainty interval for a model is the uncertainty in the expected value of the death rate. This should be much narrower than the interval for the prediction of data measurements of that expected quantity of interest. An ideal model should have data coverage close to or greater than 95%.

Some ensemble models use information on the performance of component models to generate the weights on the component models [65]. If weights are chosen from in-sample fit, these may not yield the best results. In cases where ensemble weights need to be selected on the basis of out-of-sample performance, a train-test 1-test 2 strategy needs to be used. In these cases, some percentage of the data, such as 70%, are set aside for the training set, and the remaining data are randomly split into two test datasets. The first test is used to develop ensemble weights, and the performance of all component models and the ensembles are compared using the second test dataset. In this way, the comparison in the second test between the component models and variants of ensemble models is equitable, using data that have not previously been used for any modeling strategy. Train-test 1-test 2 strategies will need to be repeated multiple times until stable performance metrics have been achieved.

#### 5. Choose the model or ensemble model with the best performance in the out-of-sample predictive validity tests

In selecting a best single model or model ensemble, the three types of assessments may have to be balanced. The model with the lowest root mean squared error (RMSE) may do worse on trend or have coverage that is too high or too low. Balancing these attributes will depend on the specific cause of death application. An overall judgment to choose the final model or model ensemble should take into account preferences for these three domains. A set of preferences should in principle be translated into an objective function that captures the desired trade-offs between different dimensions of performance. Most users will, in fact, want to balance various attributes of predictions, and they will want to examine metrics of predictive validity that capture these various attributes. In all cases, model selection should be designed to be robust to outliers.

## Methods

### Cause of Death Ensemble model (CODEm) - an integrated cause of death modeling environment

We have developed a cause of death modeling environment to facilitate work on modeling cause-specific mortality for a large number of countries, which can be applied to any cause of death for which data are available. To design this modeling tool we have developed a specific implementation of the five principles that we have outlined above. Many specific choices were required to develop a computationally tractable but flexible strategy that is consistent with these principles. In this section, we describe in detail these design choices, including the development of a large set of plausible models, the development of ensemble models using adaptive weighting systems, the assessment of out-of-sample predictive validity, and final results using maternal mortality as a case study.

We illustrate the application of CODEm to modeling several major causes of death using the cause of death database that has been developed at the Institute for Health Metrics and Evaluation. This database has been developed following the first two principles outlined above. For reference, Table 1 summarizes the available cause of death data from vital registration systems, verbal autopsy studies, surveillance systems, and various surveys/censuses with some cause-specific data. In addition, it includes data based on information collected at hospitals, mortuaries, burial sites, etc. Data inputs have been processed to deal with various issues to enhance comparability. For example, Naghavi et al. have developed algorithms to systematically deal with problems of ICD revision comparability and the phenomenon of "garbage coding"[49]. In other cases, datasets have been made comparable by mapping from aggregated age groups to five-year age groups. The end result of this work is a database of multiple sources of cause of death data that is continuously updated as new datasets are identified. While we use this database to illustrate the application of CODEm, in principle CODEm can be applied to any cause of death dataset.

**Table 1:** CODEm data sources by type and decade

Type	1980-1989	1990-1999	2000-2010	Total
**Cancer registry**	389	505	392	1286

**Other**	0	14	52	66

**Sibling history**	410	807	325	1542

**Surveillance**	3	31	55	89

**Survey/census**	1	54	49	104

**Verbal autopsy**	138	175	143	456

**Vital registration**	799	969	909	2677

### Developing plausible models

#### Model families

We have developed four families of models that reflect the choice of the dependent variable and the use of spatial-temporal patterns in the data. Table 2 shows the four families of models. One choice is whether to model cause fractions or death rates; the second choice is whether to use a simple linear hierarchical model or to also capture spatial-temporal patterns in the unexplained component of the data. As highlighted in Table 2, taking both choices into account yields four modeling families. We model cause fractions using the logit of the cause fraction. Mortality has long been shown to have an exponential relationship to units of time [28,76], so we model death rates using the natural logarithm (ln) of rates. For each family of models, we include multiple possible covariate combinations, resulting in possibly hundreds of different models.

**Table 2:** Families of CODEm component models

	Linear mixed effects model	Spatial-temporal model
**Ln(cause-specific death rate)**	1	3

**Logit(cause fraction)**	2	4

#### Choosing covariates

For each cause of death, one could postulate many feasible models based on the published literature where cohort studies, cross-sectional time series studies, or intervention trials have suggested that there is a relationship between a covariate and a specific cause of death. Because of multicollinearity, including all possible covariates in a model often yields implausible signs on covariates or unstable coefficients, as well as overfitting. There is an extensive literature on choosing covariates in these circumstances, including forward and backward stepwise regression [77,78], LASSO (L1-constrained regression) [79], elastic nets [80], Bayesian variable selection [81], and other covariate selection methods. Especially when predicting out of sample for countries with no data or very limited data, the specific choice of covariates can make a large difference on prediction [82,83]. Building on the various traditions for choosing covariates, we have developed an algorithm that captures both strong views on the plausible relationships between covariates and the relevant cause of death as well as our desire to propose a diversity of plausible models. Ideally the uncertainty of the covariates would also be taken into account, but it is not computationally feasible at present to bootstrap each covariate.

Our solution to this problem is a covariate selection algorithm that takes into account prior information on which covariates are important and what sort of effect they should have on the dependent variable. We first select *n *covariates and categorize them into three groups based on how strong the evidence is of the causal connection. For each disease, the literature was searched for previous studies showing correlations between available covariates and cause-specific mortality (particularly at the population level), and disease experts were consulted. Covariates with strong proximal relationships, such as etiological or biological roles in the disease process, are ranked as level 1. Covariates for which there is strong evidence for a relationship, but not a direct biological link, are placed in level 2. Covariates with weak evidence for a relationship, or which would be distal in the causal chain and thus may be mediated by factors in levels 1 or 2, are categorized as level 3. Based on the literature, we assign a prior on the direction of each covariate. Covariates that should increase the dependent variable are classified as having a positive prior, while those that should be inversely related are given a negative prior. If there is conflicting or inconclusive evidence as to the expected direction, the user can also specify that either direction would be valid. By definition, we would not expect to assign an ambiguous direction for a level 1 covariate.

After priors have been set according to level of evidence and presumed direction, a list of all possible covariate combinations for level 1 is created. We test all 2^n^-1 combinations of level 1 covariates. We retain all models where the sign on all covariates in that model is in the expected direction and the coefficient is significant at the p < .05 level. In each case, the model is estimated with the mixed effects structure used in the spatial-temporal model described below (random effects on super-region, region, and age), with the covariates under exploration included as fixed effects. So as to not have to retest each of these covariate combinations with the linear model family described below, we make the simplifying assumption that the inclusion of an additional country random effect will not change the direction or significance of the fixed effects; this assumption has held true in our testing and greatly reduces computational burden.

Level 2 covariates are assessed using a more computationally efficient approach. First, for each level 1 model that was retained, we create a list of 2^m ^possible level 2 models (where *m *is the number of level 2 covariates). The first, which has no level 2 covariates included, has already been tested and is retained. Next, each of the *m *possible models in which one covariate is added to the level 1 model is tested. If adding the level 2 covariate does not affect either the significance or the sign on any level 1 coefficients, and the level 2 covariate itself fits the priors on direction and significance, then it is retained as another possible model. If, however, the level 2 covariate does not fulfill the priors or forces any of the level 1 covariates to violate their priors, then it is dropped.

In addition, all other possible level 2 models that contain that covariate are also dropped. For example, if we have a level 1 model A, and three possible level 2 covariates, X, Y, and Z, we first test each of them individually against level 1 model A. Suppose × and Y fulfill the priors, but Z violates them. Then, we can eliminate the level 2 models Z, X+Z, Y+Z, and X+Y+Z. Thus, we can test just one more level 2 model, X+Y. If that model fulfills the prior, then we are left with 4 different models from our original level 1 model: A (level 1 only), A+X, A+Y, and A+X+Y. This is a conservative approach because it reduces computational burden - both for the software which thus has fewer models to evaluate, and for the analyst who typically lacks the sorts of information that would be necessary to formulate conditional priors for multiple covariates. However, if the analyst has sufficient time and computational power, all covariates can be designated as level 1 and thus all combinations of covariates will be tested.

Next, we take each of the models resulting from level 2 and use the same process as described for level 2 on the level 3 covariates. In this way, we can either test or preemptively eliminate all (2^n^-1)*2^m^*2^l ^possible covariate models (where *n *= the number of level 1 covariates, *m *the number of level 2 covariates, and *l *the number of level 3 covariates) in an efficient manner. Ultimately we obtain a set of all possible covariate combinations that fulfill our priors on covariate direction. This ensures that we do not exclude any potentially valuable information in our modeling process. We run the covariate selection tool for both cause fractions and death rates and then create both mixed effects only and spatial-temporal models for each set of chosen covariates.

This covariate selection method, which produces a list of models for which prior covariate relationship beliefs are maintained, allows for great flexibility in model choice. If a user specifies all-cause mortality as a level 1 covariate, then a classical Preston-type cause of death model would be tested. Or if there is strong reason to believe that cohort effects are prominent for a cause of death, then cohort-lagged measures could be incorporated. For instance, in the case of lung cancer an analyst could test five-year cohort lags of smoking prevalence (in which the value used to estimate lung cancer in 60 year olds in 2005 would be the smoking prevalence amongst 55 year olds in 2000), 10-year cohort lags (in which lung cancer deaths in 60 year olds in 2005 would be based on smoking prevalence amongst 50 year olds in 1995), and so forth. By using covariate selection to select a broad pool of logical models, different strategies such as these can be employed and compete on the same metrics of performance.

#### Linear mixed effects models

Model families 1 and 2 use a mixed effects model with fixed effects on covariates (selected via the mechanism explained below) and age dummies, plus hierarchical random effects by super-region, region, country, and age. The fixed effects allow us to capture broad trends in both age patterns and the impacts of key biological and environmental covariates. The random effects allow for improved estimation by adding intercept shifts by GBD super-region, region, and country (the 187 countries are grouped into 21 regions based on both geographical proximity and epidemiologic similarity; the 21 regions are further grouped into seven more general super-regions), and changes in age patterns across regions and countries. The models of this family follow this form:

$$\begin{array}{c} \ln\left(rate_{s,r,c,y,a}\right)=\beta_{i}X_{i_{s,r,c,y,a}}+\beta_{a}d+\pi_{s}+\pi_{s,r}\\ +\pi_{s,r,a}+\pi_{s,r,a,c}+\varepsilon_{s,r,c,y,a} \end{array}$$

$$\begin{array}{c} \text{logit}\left(cause\;fraction_{s,r,c,y,a}\right)=\beta_{i}X_{i_{s,r,c,y,a}}\\ +\beta_{a}d+\pi_{s}+\pi_{s,r}+\pi_{s,r,a}+\pi_{s,r,a,c}+\varepsilon_{s,r,c,y,a} \end{array}$$

Where:

s = super-region index; r = region index; c = country index; y = year index; a = age index

[countries are nested within regions, which are nested within super-regions]

β_i _= coefficient on covariate i

X_is, r, c, y, a _= covariate i for observation s, r, c, y, a

β_a _= coefficient on age offsets

d = age dummy variables

π_s _= random intercept on super-region

π_s, r _= random intercept on region (nested within super-region)

π_s, r, a _= random intercept on age (nested within region)

π_s, r, a, c _= random intercept on country (nested within region-age)

$$\varepsilon_{s,r,c,y,a}\sim \text{N(0, }\sigma_{\varepsilon }∙\text{I)}$$

Because of the small numbers that are often encountered for certain age groups, countries, or causes of death, covariate models may occasionally predict numbers that are negative in natural log or logit space. To avoid creating very large residuals that can negatively affect subsequent prediction steps, we have introduced a floor such that the predictions never go below a rate of .01 deaths per 100,000 people. In addition, log rate models, unlike logit cause-fraction models, are not constrained from predicting more deaths than the all-cause mortality rate. We have greater confidence in all-cause mortality predictions, because there are typically more data available for predicting all-cause mortality rates in the form of censuses, demographic and health surveys, vital registration systems that do not capture cause of death, etc. We have therefore placed a ceiling on log rate models such that they can never exceed the log all-cause mortality rate.

#### Spatial-temporal models

Model families 3 and 4 begin with nearly the same mixed effects model used for families 1 and 2, but with the removal of the random country effect. The spatial-temporal models then utilize additional regression analysis to take into account how the dependent variable further varies across time, space, and age. This type of spatial-temporal regression model has been used in many applications, including the estimation of maternal mortality [36]. We do this by calculating the residual (predicted - observed dependent variable) for each data point and then run local regression in three dimensions on the residual. The process assumes that residuals contain valuable information that cannot be directly observed but nonetheless vary systematically across geographic region, time, and age group. This allows us to predict how much the observed dependent variable differs from the mixed effects model's prediction and to account for these differences.

In order to perform the local regressions we must cycle through each observation in the dataset, weight every other observation in the dataset relative to it, and then find the weighted mean of the residual term. The first dimension across which we calculate weights is age. We use a simple exponential decay function

$$w_{a_{i,j}}=\frac{1}{e^{\varpi \times |agegroup_{\text{i}}-agegroup_{\text{j}}|}}$$

such that if observation *j *has the same age group as the observation we're predicting for (*i*), then it receives an age weight of 1. When ω is set to its default value of 1, if *j *is in the adjacent age group, it receives a weight of .367, if it is two age groups away the weight is .135, etc. We chose this weighting scheme recognizing that mortality estimates typically change smoothly over age. For causes with sparse data, ω can be decreased to induce greater smoothing over age; in causes of death that we expect to have very different levels by age, ω can be increased to reduce smoothing over age groups.

Next, we weight all observations *j *relative to observation *i *in time by using a weighting scheme similar to the tricubic weights used in traditional LOESS local regression:

$$w_{t_{i,j}}={\left(1-{\left(\frac{|year_{i}-year_{j}|}{\text{argmax}\left(|year_{i}\right)-\left(year_{j}|\right)+1}\right)}^{\lambda }\right)}^{3}$$

The key difference between our time weights and traditional LOESS weights is that we leave λ as a parameter that can be tuned to increase or decrease how much smoothing occurs across time. We use λ = .5 for countries that have data. If we were predicting the year 1995 in a time series from 1980 to 2010, then this would correspond to a time weight of 1 for observations from 1995, a weight of .42 for observations from 1994 or 1996, a weight of .27 for 1993 or 1997, a weight of .09 for 1990 or 2000, and a weight of close to 0 for 1980 or 2010. For countries without data, we have much less certainty in their trends; thus, we use a higher λ of 2, which smooths out predictions and avoids issues of compositional bias.

The age and time weights are multiplied together for each observation, producing a weight that reflects proximity in both dimensions. Then, the weights are rescaled to reflect geographical proximity to the observation being predicted. We use the following formula to rescale the weights:

$$w_{i,j}=\zeta \times \frac{w_{a_{i,j}}\times w_{t_{i,j}}}{\sum \left(w_{a_{i,j}}\times w_{t_{i,j}}\right)}$$

For data from country i

$$w_{i,j}=\zeta \times \left(1-\zeta \right)\times \frac{w_{a_{i,j}}\times w_{t_{i,j}}}{\sum \left(w_{a_{i,j}}\times w_{t_{i,j}}\right)}$$

For data from region i, but not country i

$$w_{i,j}={\left(1-\zeta \right)}^{2}\times \frac{w_{a_{i,j}}\times w_{t_{i,j}}}{\sum \left(w_{a_{i,j}}\times w_{t_{i,j}}\right)}$$

For data from super-region i, but not region i

w_i,j _= 0 for data from outside of super-region i

We use a value of ζ = .9 for countries with data, such that 90% of the weight in the local regression is given to observations from the same country, 9% is given to data from the same region but outside the country, and just 1% is given to data in other parts of the super-region. When there are no data for a country, we use ζ = .7, which gives 70% of the weight to data from within the region and 30% to data from other parts of the super-region; this reflects the fact that we wish to borrow more strength in such data-sparse areas.

In addition to the geographic weighting described above, we also rescale the weights inside a country when both national and subnational data are present. In cases where we have both national and subnational observations for the country we're predicting, we rescale the weights such that 90% of the in-country weight goes to the nationally representative data points and 10% is assigned to the subnational data. This reflects our desire to capture nationally representative trends.

Once these weights have been calculated, weighting every observation in the dataset relative to the one being predicted, it a simple matter of calculating a weighted average of the residuals from the mixed effects regression. This "predicted residual" is then added back onto the mixed effects prediction, creating an estimate that more closely takes into account aspects of the data that cannot be captured by a simple covariate model.

#### Gaussian Process Regression

Gaussian Process Regression (GPR) is a Bayesian estimation technique that is well-suited to estimating time series data because it maintains correlation in the uncertainty over time. The resulting estimates also track in-sample data very closely without significantly changing the predictions out of sample. We utilize GPR as the final step in our spatial-temporal component models. The inputs required are the mean function, amplitude, scale, degree of differentiability, and data variance.

Each of the models developed for the different combinations of covariates in the two spatial-temporal families provides the mean function (a prior estimate of the dependent variable). We run a separate instance of GPR for each country-age group, such that GPR will simply pick up temporal trends. Amplitude is meant to capture the uncertainty in the mean function; a higher amplitude means that the GPR will be more likely to move far away from the mean function, conditional on the data. We estimate the amplitude for each model using a robust estimate of the standard deviation of the residuals from each first stage model - specifically, we compute the standard deviation as 1.4826 times the median absolute deviation (MAD) estimate of the residuals.

The GPR parameters governing the covariance function are scale and differentiability. We assume a scale of 10 years and a differentiability of 2. In principle, these can be varied and the performance of different models tested out of sample to choose the best parameters. We have not, however, varied these as the performance of GPR in these applications does not appear to be very sensitive to the choice of these parameters.

For data variance, we would ideally like to capture variance due to sample size and sample design, as well as the data variance due to the myriad sources of nonsampling variance. Sampling variance can usually be estimated from each source on the basis of sample size and sample design. The more challenging task is to estimate the data variance due to nonsampling error. Work on child mortality and maternal mortality suggests that nonsampling variance is often substantially larger than sampling variance. In settings where multiple measurements in the same place and time period are available, it is possible to directly compute nonsampling variance. However, in most cases we have insufficient data in the same country-year to provide a direct measurement of nonsampling error.

To approximate the nonsampling error, we first compute a simple spatial-temporal weighted average of the natural log of the death rates. We then estimate for three cases the MAD estimator of the residual from this weighted average: for countries with more than 10-year sequences of vital registration data (representing systems with the highest data recording), for subnational data when national data are also present for the country, and all other cases. The MAD times 1.4826 is a robust estimate of the standard deviation [84]. The MAD estimator of the residuals includes both sampling and nonsampling error as well as the systematic variation in death rates not adequately captured by the spatial-temporal weighted average. As such it is an overestimate of nonsampling variance (NSV). We believe it is preferable to overestimate rather than underestimate data variance and have used the NSV for each data type from this procedure for all models.

#### Uncertainty estimation

In order to find uncertainty on model families 1 and 2, we first draw multiple times from the variance-covariance matrix of the fixed effects. We then simulate predicted death rates using these draws of beta, giving us an estimate of the parameter uncertainty. In order to capture the systematic variance in the model, we also add to each draw a random value from Normal(0, σ_systematic_) where σ^2^_systematic _is estimated as the square of 1.4826 times the MAD of the model residuals minus the estimated nonsampling variance.

In order to be able to capture uncertainty and the correlation structure over time in uncertainty and to be able to distinguish between uncertainty in the quantity of interest and sampling and nonsampling variance, we have used GPR [85] for both families of spatial-temporal models (3 and 4). We use the output of all covariate possibilities for the two spatial-temporal local regressions as the mean function ("prior") in GPR. Our implementation of GPR follows the approach of Rajaratnam et al. used for child and adult mortality estimation [42,86]. GPR input derivations are explained in the previous section.

#### Creating ensemble models

In addition to the component models in the four families described above, following the literature on prediction we have also created and tested ensemble models that are themselves made up from weighted draws from the posterior distribution of component models. Both theoretical and empirical findings suggest that ensemble models often have slightly better predictions but substantially more accurate assessments of prediction uncertainty [87]. One potential key criticism of cause of death predictions is that uncertainty due to model specification is not captured. Ensemble models provide a clear strategy to capture specification uncertainty in the prediction intervals.

There are multiple approaches to developing component models. Ensemble Bayesian Model Averaging (Ensemble BMA) models as proposed by Raftery, Gneiting, et al. [65] assess the probability of each model conditional on the training data. The ensemble model is then created based on weights that are equal to the probability of each model divided by the sum of the probabilities of all models. Ensemble BMA models have been found to outperform the best component model in weather forecasting, hydrology, and other fields [59,65,87,88]. Ensemble BMA weights are generally based on the performance of the model in the training data (see below for the creation of training and test datasets).

In the case of our cause of death applications, we have found that in-sample performance in the training dataset can be a poor predictor for performance out of sample in the test data. Specifically, some models with good or even the best performance in sample may yield poor performance out of sample. In these cases, it is possible to develop ensemble weights based on the probability of the model conditional on the test data, not the training data. To demonstrate objectively the performance of an ensemble weighted by the out-of-sample performance of the component models requires a strategy of splitting the data into three groups: training, a test dataset 1 used for estimating the probability of the model conditional on the test data, and a test dataset 2 to assess performance of the component models and ensembles based on out-of-sample performance.

Based on the Netflix challenge [54] and other prediction efforts, other ensemble strategies may also perform well. Simple averaging of all plausible models is one possibility. Simple averages of all models or of the top performing models can avoid adding to the variance in the model due to misestimation of the weights on each component model. A compromise approach is to average the top *X *models, which effectively puts 0 or 1/*X *weights on each component model. Another option is to use a monotonically declining function for weights based on the ranked performance of each component. This approach provides some reward for better performance but does not try to directly estimate a weight from the data. One example of a monotonically declining weight on each component model is to use the function:

$$W_{i}=\frac{\psi^{\left(N-rank_{i}\right)}}{\sum_{j=1}^{N}\left(\psi^{\left(N-j\right)}\right)}$$

**Figure 2:**
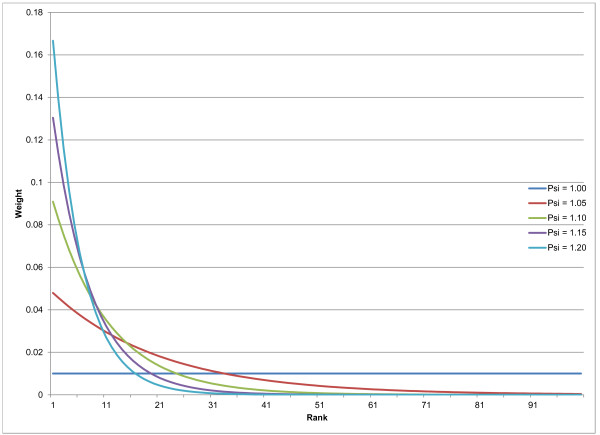
**Example weighting schemes for 100 component models at different values of ψ**.

Where *N *is the number of models and ψ is a parameter influencing the relative weighting of models. In this case, if there were four total models to choose from and ψ = 1.2, the top performing model would be given 32.2% weight, the second model 26.8%, the third 22.4%, and the last 18.6%. We use the weights to determine how many draws from each model to add to the final pool. We take 1,000 draws overall, so the top performing model would have 533 draws included in the average in this example, whereas the worst performer would have only 67 draws contributing to the final average. Figure 2 demonstrates how the weighting function works for different values of ψ in the case of 100 component models.

In an ensemble of 100 possible component models, a ψ of 1.2 would result in 167 draws from the top model, 80 for the fifth place model, 32 from the 10^th ^place model, two from the 25^th ^place model, etc., and no draws at all from models ranked below the top 32 (effectively placing a threshold such that the bottom 68% of models in this case have no bearing on the final predictions). In contrast, an ensemble with ψ = 1 would take equal draws from all models.

We develop and test ensembles that include ψ values from 1.0 to 1.2 in intervals of .01. We can then compare these different ensemble models using predictive validity performance on the second set of test data. This enables us to choose an ensemble model that weights the component models adaptively. An ensemble with some good models and some very bad models would perform best with a higher ψ value; perhaps surprisingly, previous studies have shown that an ensemble with all models having similar performance often performs best when all models are included (i.e., in the case of CODEm, when a lower value of ψ[55] is chosen).

Because the component models and ensembles are developed without any access to the second test dataset, the evaluation of performance of each strategy is based on a fair out-of-sample test.

### Assessing predictive validity

Our approach to predictive validity can be divided into two distinct parts: the strategy for developing train and test datasets, and the metrics used for assessing predictive validity.

#### Developing train-test 1-test 2 datasets

**Figure 3:**
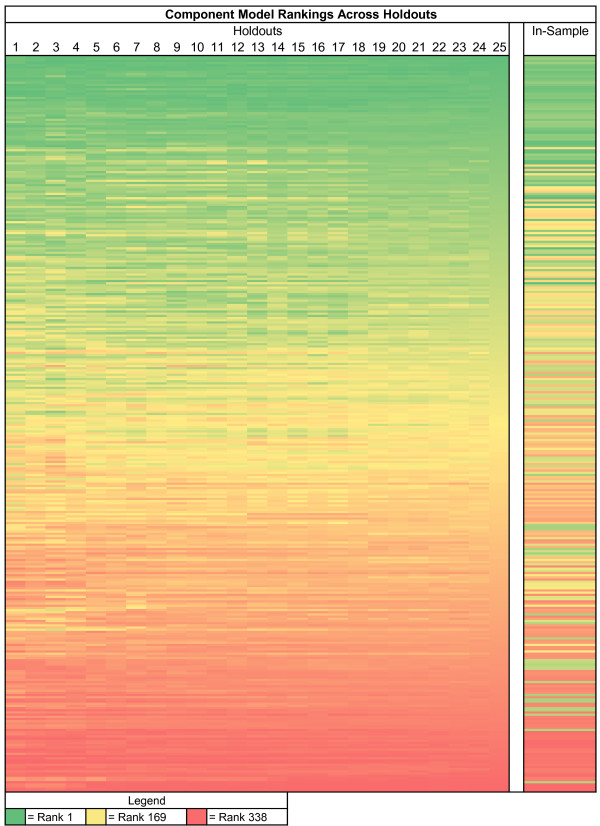
**Heatmap of stability in maternal mortality component model rankings**. Each row represents a component model, and each column corresponds to how many hold-outs are used when ranking. Rows are sorted by rankings using all 25 hold-outs. Cells are colored based on their ranking, with the green models being the best and red the worst. The last column displays in-sample ranks. The figure shows that rankings stabilize as hold-outs increase, and that in-sample ranking does not correlate well with out-of-sample.

As noted above, when developing single models and ensemble models, we need to create datasets where the original data are split into three components: the train dataset, the test 1 dataset, and the test 2 dataset. While much of the literature in bioinformatics uses 80-20 splits of the data when there is only one test set, we have compromised and used 70% of the data for training the models, 15% for test 1, and 15% for test 2. We have found that results from this type of "knockout" strategy provide stable model orderings when the process is repeated 10 to 30 times. Figure 3 demonstrates that component model rankings on the test 1 dataset are susceptible to noise when there are just a few hold-outs but begin to stabilize after approximately 10 hold-outs. Furthermore, it demonstrates that in-sample fit is a poor predictor of out-of-sample fit.

Our knockout pattern operates as follows: we create two randomly shuffled lists of all 187 countries, × and Y. We take the first country in list × and mark for which age-year pairs it is missing data for the selected cause and sex. We then knock out any data that are present in those age-years for the first country from list Y. For instance, if the country from list × had a missingness profile like that of Scenario D in Figure 1 (in which data after 2000 are not present) and the list Y country had all age-year pairs present, then we would knockout the data from 2001 to 2010 for the list Y country, leaving in the data from 1980 to 2000. We repeat this process for pairs of countries until 15% of the data in list Y are knocked out, assigning those data points to the test 1 set. We then continue down the list of country pairs until an additional 15% of the data have been knocked out, which we assign to test 2. Note that this test is excessively stringent, because it does not take into account whether or not the country from list Y actually contains data in the same age-years as the list × country, making the task harder in most cases.

We create 25 sets of train-test-test datasets based on the knockout strategy just described, then run all the component models on the training data. We use the results of each component model to predict for the test 1 data. We then assess the performance of each component model in the test 1 dataset using the metrics described below. Ensemble model weights are estimated based on the performance of component models in test 1 data. Then the ensemble models are evaluated for 21 values of ψ (1.0 to 1.2 at intervals of .01) on the test 1 data. The ensemble weighting scheme that achieves the best predictive validity metrics on test 1 is then used to create the final predictions. Its predictive validity is assessed along with the component models on the test 2 data.

#### Metrics of predictive validity

Predictive validity is evaluated using three metrics. First, we evaluate how well each model for a particular cause of death predicts age-specific death rates using the RMSE of the natural log of the death rate. Log death rates are generally comparable across age groups, so the choice of RMSE of the natural log of death rates means that we are equally concerned about a 10% error in one age group compared to another. Metrics that focus on the absolute error would tend to give highest weight to model fit in the age groups with higher rates, but most users of cause of death data actually attach greater importance to accuracy at younger ages where each death represents more years of life lost. We have thus chosen a method of assessing accuracy that we think balances the need to develop models that predict both an accurate overall level of mortality as well as an accurate age pattern. Second, we also desire models that predict accurate trends. To do this, for the test data we compute where possible the log death rate in year t minus the log death rate in year t-1. We also compute the same metric for the prediction. We then count the percentage of cases for which the model predicts a trend in the same direction as the test data.

In order to compare the performance of multiple families of models that may use different measures and transformations of cause of death data (e.g., logit cause fractions versus log death rates in our case), we must choose a single space in which to assess all the models. We have chosen to assess performance in log rate space. We prefer to assess accuracy of rates instead of cause fractions because they are generally more useful from a public health perspective - for instance, a declining rate represents progress on combatting a cause of death, whereas a declining cause fraction could result from a stagnating death rate for one cause but an increase in another. Log rates are used because of the well-established log-linear relationship with death rate and time [28,76].

Ideally, we would like to use out-of-sample coverage as an additional metric in comparing component models' performance. Unfortunately, it is computationally intractable to compute full confidence intervals for every single component model once the model pool becomes large. As computational speed improves, coverage can be added into the model ranking scheme. In the case of CODEm, we find the computationally expedient expectation of each component model for the ranking process.

We rank each component model on the median of these two metrics across hold-outs (lower being better for RMSE, higher being better for the trend test). We then sum up the ranks across the two metrics for each component model, and assign overall rank 1 to the model with the smallest sum of ranks, rank *N *to the model with the highest sum of ranks, etc. These ranks are then used in the ensemble weighting equation described above.

We similarly rank the ensemble models for different values of ψ on these two metrics and compute the sum of their ranks across hold-outs for test 2. We choose the value of ψ that minimizes the sum of ranks and use it to make our final set of predictions.

Finally, an important property of the models is that they generate plausible prediction intervals. We therefore compute the percent of data in the test set that were included in the 95% prediction interval. The prediction interval is based both on the uncertainty in the predicted death rate and the data variance for each observation. We report the mean value of each of these metrics across the 25 test 2 datasets.

## Results

We demonstrate the logic of CODEm and the performance of various models for maternal mortality. Data availability for maternal mortality are summarized in Table 3. Overall, there are 4,563 site-years available for analysis. In addition, there are a number of applicable covariates available for analysis. Table 4 provides the class 1, 2, and 3 covariates for maternal mortality, along with the priors placed on the direction of their coefficients.

**Table 3:** CODEm data sources for maternal mortality, by source type and decade

Type	1980-1989	1990-1999	2000-2010	Total
**Other**	0	5	41	46

**Sibling history**	410	807	325	1542

**Surveillance**	3	23	51	77

**Survey/census**	1	9	41	51

**Verbal autopsy**	49	72	87	208

**Vital registration**	799	944	896	2639

**Table 4:** Covariate priors for maternal mortality covariate selection

Covariate	Level	Prior
**In-facility delivery coverage**	1	-

**Skilled birth attendance coverage**	1	-

**Malnutrition (proportion under 2SD)**	1	-

**Ln(total fertility rate)**	1	+

**Age-specific fertility rate**	1	+

**Health system access score**	2	-

**Antenatal clinic (four visits) coverage**	2	-

**Antiretroviral (ARV) adjusted HIV prevalence**	2	+

**Ln(neonatal death rate)**	2	+

**Education (year per capita)**	3	-

**Ln(lag-distributed income per capita)**	3	-

The process of running covariate selection for the four families of models yields a total of 338 models, as shown in Additional File 1 for maternal mortality. In order to find these models, the covariate selection tool began with 1,984 possible models each for rates and cause fractions. We ran 261 regressions on rates and 226 on cause fractions and found 98 rate models and 71 cause fraction models that fulfilled all of our priors, each of which was tested as both a simple mixed effects model and a spatial-temporal model. The difference between 1,984 possible combinations of covariates and the 261 for which regressions were run relates to the algorithm for class 2 and class 3 covariates where models are not evaluated if the simpler model with a covariate does not have the sign and statistical significance expected.

**Figure 4:**
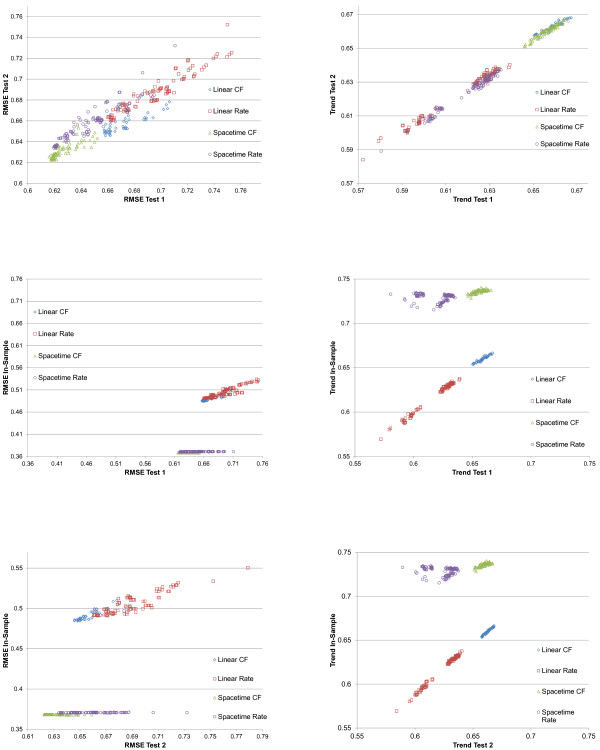
**In-sample, test 1, and test 2 performance on RMSE and trend test for maternal mortality component models**.

**Figure 5:**
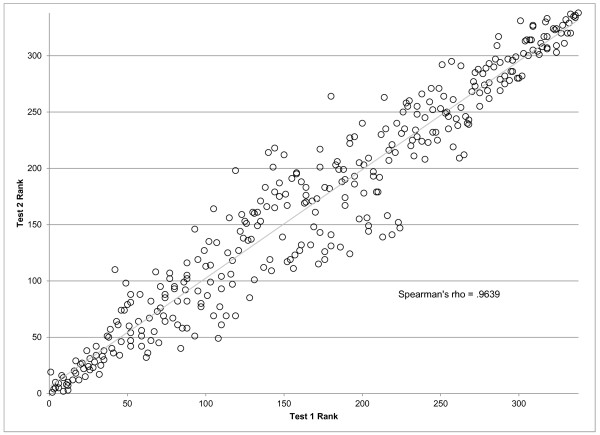
**Correlation between test 1 and test 2 rankings of maternal mortality component models**.

As seen in Figure 4, it is evident that performance varies widely across models; RMSE ranges from 0.616 to 0.774 across component models in test 1, while the trend test ranges from 0.572 to 0.667. In-sample fit ranges from 0.368 to 0.550 in RMSE and 0.569 to 0.740 on the trend test. The performance on test 2 is very similar to that of test 1, as also seen in Figure 5, suggesting that model performance is not a function of the particular hold-outs used for the assessment. Figure 4 shows several important patterns. First, for RMSE, in-sample fit for all the spatial-temporal GPR models, whether for cause fractions or for rates, are much better than for the mixed effects models. This is to be expected given that these models tend to capture some of the patterns in the residuals that are structured over space and time. However, in the case of maternal mortality, the figure also shows that in the out-of-sample RMSE tests, these models tend to also do better, although there is clear overlap between classes of models. Of note, within a class of models, particularly the spatial-temporal GPR models, there is little relationship between in-sample fit and out-of-sample RMSE. This visual impression is confirmed by the rank order correlation coefficient within model class between in-sample RMSE and out-of-sample RMSE on test 2, which are -0.369, 0.463, 0.887, and 0.830 for spatial-temporal cause fractions, spatial-temporal rates, linear cause fractions, and linear rates, respectively. The strongest relationship observed between in-sample performance and out-of-sample predictive validity is for linear cause fraction models. However, even a rank order correlation coefficient of 0.887 within that class of models means that some poorly performing models might be selected if selection is based only on in-sample fit. For the better performing classes of models, in this case the spatial-temporal cause fraction and rates models, in-sample fit is not a useful guide for model choice at all.

Figure 4 also shows a similar analysis of performance for the trend test, but the patterns are distinctly different. In the case of maternal mortality, in sample the spatial-temporal models do better than the linear models. However, out of sample, the cause fraction models perform substantially better than the rates models. In fact, in both test 1 and test 2 cases, the linear cause fraction models have on average slightly better performance than the spatial-temporal models for cause fractions; there is however substantial overlap in these sets of models on this test. Within model class, the rank order correlation coefficient for the trend test is 0.675 for spatial-temporal cause fractions, -0.174 for spatial-temporal rates, 0.975 for linear cause fractions, and 0.984 for linear rates.

**Figure 6:**
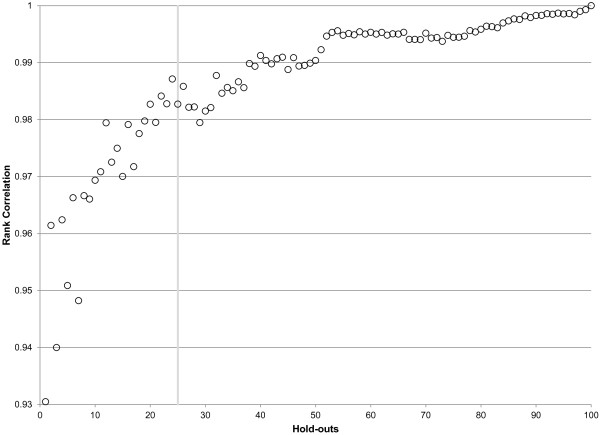
**Correlation in maternal mortality component model rankings by number of hold-outs performed**.

The high correlation of out-of-sample performance for RMSE between test 1 and test 2 provides reassurance that the ordering of models is not specific to the particular set of hold-outs used. Nevertheless, it is important to ascertain how many hold-outs are required to get a stable assessment of the ordering of different models within and between classes. Figure 6 illustrates how the stability in component model rankings improves as more hold-outs are added. After 25 hold-outs, the rank correlation exceeds .98; 40 hold-outs yields rank order correlation coefficients in excess of 0.99 for maternal mortality. Figure 3 shows that for maternal mortality the top ranked models have all become rather stable after 25 hold-outs; it further demonstrates that in-sample fit is poorly correlated with out-of-sample predictive validity. Extra hold-outs tend to further stabilize the ranking of the poorer performing models rather than change the order of the top models in this case.

Notice in Additional File 1 that spatial-temporal models on the logit of the cause fraction occupy the top 88 slots out of 338 total component models. This indicates that spatial-temporal models do indeed do a better job of capturing levels and trends not present in covariates alone. The fact that cause fraction models perform better than rate models suggests that cause fractions, which are constrained to be less than the all-cause mortality rate, may benefit from the additional information provided by being multiplied by the all-cause mortality rate when calculating final predictions. The covariates for the best ranked model included the natural log of the total fertility rate, skilled birth attendance coverage, and the natural log of lag-distributed income per capita. Similar models with additional covariates, such as the fourth-ranked model, which added age-specific fertility rates, performed worse in many cases, suggesting that while additional covariates may improve in-sample fit they often cause overfitting that harms out-of-sample predictions.

Table 5 shows how well ensemble models of varying levels of ψ perform on test 1 predictive validity. In the case of maternal mortality a ψ of 1.17 has been chosen. This is a rather steep weighting scheme, giving over half the weight to just the top five component models - the top component model receives 139 draws, relative to 102 draws for the third-place model, 46 for eighth place, 15 for 15^th ^place, etc (see the last column of Additional File 1). Interestingly, ψ of 1.0 - which gives equal weights to all models - performed the worst on both predictive validity tests, suggesting that there was at least one bad component model that would harm predictions if included.

**Table 5:** Ranking and predictive validity metrics for maternal mortality for different values of ψ

Rank	ψ	RMSE (in-sample)	RMSE (test 1)	Trend test (in-sample)	Trend test (test 1)
**1**	1.17	0.368	0.617	0.737	0.737

**2**	1.16	0.368	0.617	0.737	0.737

**3**	1.15	0.368	0.617	0.737	0.737

**3**	1.11	0.369	0.617	0.737	0.737

**5**	1.08	0.369	0.616	0.737	0.737

**6**	1.12	0.368	0.617	0.737	0.737

**7**	1.18	0.368	0.617	0.737	0.737

**8**	1.06	0.370	0.616	0.736	0.736

**9**	1.03	0.376	0.615	0.734	0.734

**10**	1.04	0.373	0.615	0.735	0.735

**11**	1.09	0.369	0.616	0.737	0.737

**12**	1.05	0.371	0.616	0.736	0.736

**13**	1.1	0.369	0.617	0.737	0.737

**14**	1.14	0.368	0.617	0.737	0.737

**14**	1.13	0.368	0.617	0.737	0.737

**16**	1.19	0.368	0.617	0.737	0.737

**17**	1.02	0.381	0.614	0.732	0.732

**18**	1.2	0.368	0.617	0.737	0.737

**19**	1.07	0.369	0.616	0.737	0.737

**20**	1.01	0.390	0.614	0.726	0.726

**21**	1	0.412	0.624	0.709	0.709

Table 6 provides the performance on test 2 of the best component model (the spatial-temporal model on logit(cause fraction) with covariates of age-specific fertility rate, the natural log of total fertility rate, and the natural log of lag-distributed income per capita) and of the best ensemble model. Results are consistent with previous findings that out-of-sample predictive validity metrics are improved by creating an ensemble of models instead of just using the top component model. RMSE decreases, trend test performance remains about the same, and the prediction interval coverage also increases.

**Table 6:** Predictive validity measures for final CODEm maternal mortality model

Model	RMSE (in-sample)	RMSE (test 2)	Trend test (in-sample)	Trend test (test 2)	Coverage (in-sample)	Coverage (test 2)
**Top ensemble**	0.368	0.626	0.739	0.665	99.2%	97.3%

**Top component**	0.369	0.636	0.737	0.663	99.2%	96.9%

Performance of the top component model is also shown for comparison.

Finally, we checked the test-retest stability of our method by running the same model with 25 hold-outs twice on maternal mortality. The Pearson correlation coefficient for the log rates of the final mean estimate (by country, year, and age) was 0.9998 for the two runs, indicating that the method gives consistent results with 25 random hold-outs. Nevertheless, given that the hold-out process and assessment of predictive validity is stochastic by nature, the exact set of top performing models for a cause of death can vary between model runs even if the predictions are highly consistent.

Table 7 shows summary results comparing the ensemble model to the best component model for several causes of death, including cardiovascular disease, chronic respiratory disease, cervical cancer, breast cancer, and lung cancer. The table highlights that in each case, to a varying degree, the ensemble model has as good as or slightly better predictive validity metrics than the component model. In most cases the out-of-sample performance for RMSE and trend test both improve or remain roughly equal when comparing the ensemble to the top component model. The one exception is lung cancer in males, in which the ensemble model has slightly worse RMSE and trend; however, the coverage of the ensemble models is much better than that of the component, so this is most likely a desirable trade-off. In no case do we observe that the ensemble model performs significantly worse than the top component model, and in many instances the ensemble is a substantial improvement. In all cases, the uncertainty interval coverage is better for the ensemble model than the best component model.

**Table 7:** Predictive validity measures for CODEm results for several selected causes of death

Cause	Model	RMSE (in-sample)	RMSE (test 2)	Trend test (in-sample)	Trend test (test 2)	Coverage (in-sample)	Coverage (test 2)
**Cardiovascular disease (male)**	Ensemble	0.123	0.290	0.764	0.699	0.993	0.853
	
	Best component model	0.122	0.292	0.764	0.693	0.992	0.822

**Cardiovascular disease (female)**	Ensemble	0.162	0.304	0.750	0.698	0.993	0.903
	
	Best component model	0.162	0.305	0.751	0.707	0.993	0.877

**Maternal mortality**	Ensemble	0.368	0.626	0.739	0.665	0.992	0.973
	
	Best component model	0.369	0.636	0.737	0.663	0.992	0.969

**Cervical cancer**	Ensemble	0.528	0.944	0.667	0.593	0.988	0.938
	
	Best component model	0.489	0.951	0.678	0.587	0.982	0.912

**Breast cancer (female)**	Ensemble	0.438	1.105	0.678	0.572	0.985	0.923
	
	Best component model	0.428	1.129	0.675	0.570	0.981	0.875

**Lung cancer (male)**	Ensemble	0.359	0.769	0.714	0.599	0.993	0.841
	
	Best component model	0.330	0.759	0.738	0.617	0.993	0.800

**Lung cancer (female)**	Ensemble	0.383	0.734	0.705	0.580	0.995	0.932
	
	Best component model	0.371	0.756	0.738	0.597	0.994	0.897

## Discussion

In this paper, we have proposed a framework for cause of death model development. We have illustrated this approach for several causes of death including maternal mortality, cardiovascular disease, chronic respiratory diseases, cervical cancer, breast cancer, and lung cancer mortality. Our strategy yields an ensemble model with smaller error in estimated rates than the single best model. It yields more accurate trends and often better uncertainty intervals with nearly 95% data coverage in out-of-sample prediction tests. We have used maternal mortality in this paper to illustrate the results of CODEm, but we have applied this strategy to many causes with similar findings; in addition, separate papers utilizing this method include more detailed results for maternal mortality, breast cancer, and cervical cancer [89,90]. The model development strategy we have used has already been widely applied in other fields; what we have done is to develop a pragmatic implementation of these ideas to cause of death modeling.

Debate on cause of death estimation, such as for maternal mortality [36,38-41], can be traced to three components: the database used, including covariates; the processing of data to enhance comparability and quality; and model building. By encompassing a very wide range of models and model families combined with objective assessment of performance through out-of-sample predictive validity tests, we expect that our approach will substantially decrease debate around model building. Debate in the future will more likely focus on the data processing step. With regards to the database, most analysts will agree that a more systematic collation of all data sources is preferable to fewer data sources. The real issue is how one uses or modifies observations in the largest database. Controversy will remain on how to deal with misclassification in cause of death assignment, subnational and nonrepresentative samples, and the exclusion of data points due to extreme nonsampling variance. We hope that by proposing and implementing a structured approach to model building, the idiosyncratic aspect of final model choice will be minimized or even eliminated. Debate on data processing will continue and hopefully be fertile ground for methods innovation in the future

In various conferences following the release of the maternal mortality studies in 2010 [36,38,39], some commentators argued that simple models should be preferred to more complex models such as GPR. Underlying these comments are two possible perspectives. First, simple or parsimonious models may actually generate better predictions out of sample. Given that we test all models, whether simple, more complicated, or ensemble on a level playing field, if simple models are better they will be selected as the basis for making predictions in our modeling framework. The second possible interpretation of this view is more philosophical, namely that simple models are intrinsically preferable regardless of predictive performance. In other fields, like weather forecasting, consumers do not demand simple models; they demand accurate models. Not surprisingly, weather forecasters - who have in some ways an easier task than cause of death estimation, as their forecasts are shorter and have much more data to rely on - use dynamic ensemble models where the weights on various models are selected each day for tomorrow's forecast [87] in order to generate predictions with the smallest error and accurate uncertainty intervals. This is entirely inconsistent with a call from some in global health to sacrifice performance of cause of death models for the sake of simplicity. In this era, we all use complex devices such as computers, cell phones, or even watches. Most of us do not understand how the circuitry or machinery within the device works. We judge these devices by how well they perform, not how well we understand their inner workings. We believe that the same ethos ought to be applied for cause of death modeling. The predictive validity metrics we have described in this paper are useful not only for ranking researchers' own models and creating ensembles, but we believe they can also provide a framework within which modeling strategies from diverse research groups can be fairly compared. Since it is good out-of-sample predictive performance that should ultimately decide which models are most useful, then we think models should be evaluated and compared on those grounds.

The current implementation of our strategy has several limitations. The Netflix challenge experience suggests that pooling results from very different modeling strategies performs better [54]. We have used four modeling families that are the product of modeling natural log rates and logit cause fractions, mixed effect linear models, and spatial-temporal GPR models. This creates a diverse pool of models that compete in predictive validity tests. For example, combined with the covariate selection approach, we had in the case of maternal mortality 7,936 possible component models. Nevertheless, many other modeling strategies are possible; given the experience in other areas, expanding the universe of modeling families that are included in the model pool will likely improve prediction performance. The fundamental challenge to expanding beyond our four families of models is computational power and time. In our implementation, we have already reached the limit of what is currently practical - each model run takes approximately 600 hours of processor time, which for our cluster is equal to about 5,000 GFlops. As computational power and cloud computing get progressively cheaper and estimation algorithms more and more optimized, it may be possible in future iterations to increase the diversity of the model pool and therefore the prediction performance of the model ensemble. As with any modeling strategy, the approach outlined here depends critically on the database that is used for estimation, the quality of the covariates, and the assessment of the evidence on the expected relationships for covariates.

Although model building is becoming a computationally intensive but replicable and less subjective task, the challenge remains of capturing uncertainty due to correcting data for bias, excluding data points as outliers, and measuring and forecasting covariates. Garbage code redistribution algorithms are largely based on expert opinion, although some empirical approaches have been applied [51]. Methods to capture uncertainty in these approaches need to be developed. For verbal autopsy, the results of the Population Health Metrics Research Consortium verbal autopsy studies [91,92] provide a potential strategy for assessing misclassification uncertainty. For outliers or observations with high nonsampling variance, one can imagine a number of strategies. Instead of assigning 0 or 1 weights to a data point, 0 if the point is an outlier and 1 if it is not, numbers from 0 to 1 could be assigned that represent the probability that a data point is an outlier. Repeated sampling of the dataset could be undertaken using this matrix, allowing for propagating uncertainty. Alternatively, outliers could be assigned higher nonsampling variance in techniques such as GPR that can take this into account. The covariates we use as inputs to our models also have measurement error, and because they are often themselves modeled they may also have prediction uncertainty. It is unclear if incorporating this uncertainty would be helpful in predicting causes of death since we test out-of-sample data coverage to check that it is close to 95%, but because of computational limitations we have been unable to test the usefulness of including such information. These approaches all represent attractive avenues for further exploration. The main limitation is the computational time and cost that such schemes would imply.

In many cases, cause of death information is most valuable when placed into context with the entire cause of death composition of the population. The relative burdens of causes of death are often more influential in priority setting than are the individual causes' respective sizes. For this reason, it is often desirable to produce estimates of mutually exclusive and collectively exhaustive cause of death categories. Ideally, one would design a model that predicts for all causes of death simultaneously, accounting for correlations and dependencies between them. There are, however, three major limitations on modeling all causes simultaneously: severe restrictions on model diversity, limitations on data usability, and infeasibility for analysis.

First, current computational restrictions make it intractable to fit multinomial or seemingly unrelated regression models while still utilizing the wealth of spatial-temporal information present in the datasets. We have found that borrowing strength across space, time, and age groups in the data has substantially improved predictive accuracy. Current techniques do not allow for us to take advantage of such modeling advances within a multinomial framework, unfortunately; as computational power continues to increase and Bayesian sampling methods for highly multidimensional problems improve, such a model will likely be feasible in the future.

Second, for many causes of death such as maternal mortality, there are extensive datasets that provide measurements of only one cause of death. For example, modeling all causes of death of reproductive aged women simultaneously would limit the use of these rich sources of data. The same situation exists for many specific causes of death where there are cause-specific datasets, such as population-based cancer registries that do not include cause of death information for a set of mutually exclusive and collectively exhaustive causes. Finally, we believe it is infeasible for all studies of causes of death to always examine all causes simultaneously. Researchers will legitimately want to investigate single causes of death at a time in addition to more comprehensive studies covering all causes.

For those cases such as the GBD Study, where estimates are undertaken for a set of mutually exclusive and collectively exhaustive cause of death categories, we recommend a two-step process. First, produce models with the best out-of-sample predictive validity for each cause of death independently, taking advantage of all available high quality data and capturing spatial-temporal patterns in the data. Second, predicted cause of death estimates for each cause can be modified to sum to the total all-cause mortality predictions. This might be desirable because there are substantially more data available for all-cause mortality, meaning we have more confidence in these predictions of total mortality than we would in simply combining all the single cause models together and taking the sum to be all-cause mortality. The most appropriate methods for combining estimates of cause-specific mortality in a country-sex-age group to equal the all-cause mortality estimates that appropriately take into account the variance of different predictions by cause is a topic of active research beyond the scope of this study. While such an approach is not ideal, it outperforms models that make the trade-off of using much more restrictive assumptions in order to fit within a framework that models all causes simultaneously. Demonstrations of the overall predictive validity of the two-step approach applied to selected cases will be an important area for future research.

Our model building strategy implemented in CODEm and alternative implementations that will surely come in the future all require access to substantial computational power. Cheaper cloud computing and processors mean that many research groups around the world already have access to the required computational resources. These groups will likely pursue even more sophisticated approaches in the coming years that have more diverse model pools and capture uncertainty that stems from data processing. But for many users, even the current strategies will be beyond their available computational power. We believe that it will be important to catalyze access to these approaches for users especially in the developing world. Through internet-accessed servers dedicated to this type of processing, it should be possible to find a feasible way to make these tools more widely available.

No doubt, vigorous debate on the level and trend of important causes of death such as maternal causes, malaria, and cardiovascular diseases will continue. Such debates will continue even in high-income countries with complete vital registration and medical certification of causes of death because of misclassification biases. Cause of death estimation will continue to rely on appropriate modeling strategies to better understand real, underlying trends in diseases and injuries. Consensus around a set of principles for cause of death model building will be of great and immediate value to the global public health community who rely on this information for monitoring progress with development goals. CODEm is one pragmatic implementation of the principles laid out in this paper, one that will have extensive use in the GBD 2010 Study. More widespread implementation of these principles will hopefully continue to emerge over the next few years, leading to a much better understanding of levels and trends in key causes of death.

## Competing interests

The authors declare that they have no competing interests.

## Authors' contributions

CM led the project, including establishing the principles of model development, framing the statistical approach, interpreting results, and writing the first draft of the paper. KF developed the statistical methods, designed the software application, interpreted results, and wrote the second draft of the paper. RL and AL gave analysis and interpretation throughout the development process to shape the final form of the methods. All authors read and approved the final version of the manuscript.

## Supplementary Material

Additional file 1 **Descriptions, rankings, and predictive validity metrics for maternal mortality component models**. Click here for file

## References

[B1] Mathers CD Fat DM Inoue M Rao C Lopez AD Counting the dead and what they died from: an assessment of the global status of cause of death data Bull World Health Organ 2005 83 171 177 15798840 PMC2624200

[B2] Sibai AM Mortality certification and cause-of-death reporting in developing countries Bull World Health Organ 2004 82 83 83 15042227 PMC2585897

[B3] Ruzicka LT Lopez AD The use of cause-of-death statistics for health situation assessment: national and international experiences World Health Stat Q 1990 43 249 258 2293493

[B4] Gakidou E Mallinger L Abbot-Klafter J Guerrero R Villalpando S Lopez Ridaura R Aekplakorn W Naghavi M Lim S Lozano R Murray CJ Management of diabetes and associated cardiovascular risk factors in seven countries: A comparison of data from national health examination surveys Bulletin of the World Health Organization 2011 89 172 183 10.2471/BLT.10.080820 21379413 PMC3044248

[B5] Danaei G Finucane MM Lu Y Singh GM Cowan MJ Paciorek CJ Lin JK Farzadfar F Khang Y-H Stevens GA Rao M Ali MK Riley LM Robinson CA Ezzati M National, regional, and global trends in fasting plasma glucose and diabetes prevalence since 1980: systematic analysis of health examination surveys and epidemiological studies with 370 country-years and 2·7 million participants The Lancet 2011 378 31 40 10.1016/S0140-6736(11)60679-X 21705069

[B6] Preston S Kevfitz N Schoen R Causes of death Life tables for national populations 1972 New York: Seminar Press

[B7] Jougla E Pavillon G Rossollin F De Smedt M Bonte J Improvement of the quality and comparability of causes-of-death statistics inside the European Community Rev Epidemiol Sante Publique 1998 46 447 56 9950045

[B8] Glasser JH The quality and utility of death certificate data Am J Public Health 1981 71 231 233 10.2105/AJPH.71.3.231 7468853 PMC1619790

[B9] World Health Organization Manual of the International Statistical Classification of Diseases, Injuries, and Causes of Death, 1975 Revision 1977 Geneva: World Health Organization

[B10] World Health Organization International statistical classification of diseases and related health problems, 10th revision 1992 Geneva: World Health Organization

[B11] Anderson RN Miniño AM Hoyert DL Rosenberg HM Comparability of cause of death between ICD-9 and ICD-10: preliminary estimates Natl Vital Stat Rep 2001 49 1 32 11381674

[B12] Jemal A Ward E Anderson RN Thun MJ Influence of Rules From the Tenth Revision of the International Classification of Diseases on U.S. Cancer Mortality Trends Journal of the National Cancer Institute 2003 95 1727 1728 10.1093/jnci/djg116 14625267

[B13] Rooney C Griffiths C Cook L The implementation of ICD-10 for cause of death coding-some preliminary results from the bridge coding study Health Statistics Quarterly 2002 13 31 41

[B14] Grigg B Brooks RG Lieb S Grigg M Coding Changes and Apparent HIV/AIDS Mortality Trends in Florida, 1999 JAMA: The Journal of the American Medical Association 2001 286 1839 10.1001/jama.286.15.1839 11597284

[B15] Yudkin PL Burger EH Bradshaw D Groenewald P Ward AM Volmink J Deaths caused by HIV disease under-reported in South Africa AIDS 2009 23 1600 1602 10.1097/QAD.0b013e32832d4719 19521232

[B16] Groenewald P Nannan N Bourne D Laubscher R Bradshaw D Identifying deaths from AIDS in South Africa AIDS 2005 19 193 201 10.1097/00002030-200501280-00012 15668545

[B17] Kern EFO Maney M Miller DR Tseng C Tiwari A Rajan M Aron D Pogach L Failure of ICD-9-CM Codes to Identify Patients with Comorbid Chronic Kidney Disease in Diabetes Health Services Research 2006 41 564 580 10.1111/j.1475-6773.2005.00482.x 16584465 PMC1702507

[B18] D'Amico M Agozzino E Biagino A Simonetti A Marinelli P Ill-defined and multiple causes on death certificates - A study of misclassification in mortality statistics European Journal of Epidemiology 1999 15 141 48 10.1023/A:1007570405888 10204643

[B19] Cheng WS Wingard DL Kritz-Silverstein D Barrett-Connor E Sensitivity and Specificity of Death Certificates for Diabetes Diabetes Care 2008 31 279 284 17959866 10.2337/dc07-1327 PMC2654202

[B20] Lu T-H Anderson RN Kawachi I Trends in Frequency of Reporting Improper Diabetes-related Cause-of-Death Statements on Death Certificates, 1985-2005: An Algorithm to Identify Incorrect Causal Sequences American Journal of Epidemiology 2010 171 1069 1078 10.1093/aje/kwq057 20413407

[B21] McEwen LN Karter AJ Curb JD Marrero DG Crosson JC Herman WH Temporal Trends in Recording of Diabetes on Death Certificates Diabetes Care 2011 34 1529 1533 10.2337/dc10-2312 21709292 PMC3120163

[B22] Morton L Omar R Carroll S Beirne M Halliday D Taylor K Incomplete and inaccurate death certification - the impact on research Journal of Public Health 2000 22 133 137 10.1093/pubmed/22.2.133 10912549

[B23] Sehdev AES Hutchins GM Problems With Proper Completion and Accuracy of the Cause-of-Death Statement Arch Intern Med 2001 161 277 284 10.1001/archinte.161.2.277 11176744

[B24] Lahti RA Penttilä A The validity of death certificates: routine validation of death certification and its effects on mortality statistics Forensic Sci Int 2001 115 15 32 10.1016/S0379-0738(00)00300-5 11056267

[B25] Mackenbach JP Van Duyne WM Kelson MC Certification and coding of two underlying causes of death in The Netherlands and other countries of the European Community Journal of Epidemiology and Community Health 1987 41 156 160 10.1136/jech.41.2.156 3655636 PMC1052602

[B26] Lakkireddy DR Gowda MS Murray CW Basarakodu KR Vacek JL Death certificate completion: How well are physicians trained and are cardiovascular causes overstated? The American Journal of Medicine 2004 117 492 498 10.1016/j.amjmed.2004.04.018 15464706

[B27] Lloyd-Jones DM Martin DO Larson MG Levy D Accuracy of Death Certificates for Coding Coronary Heart Disease as the Cause of Death Annals of Internal Medicine 1998 129 1020 1026 9867756 10.7326/0003-4819-129-12-199812150-00005

[B28] Preston SH Mortality Patterns in National Populations: With Special Reference to Recorded Causes of Death 1976 New York: Academic Pr

[B29] Lopez AD Hull TH A note on estimating the cause of death structure in high mortality populations Popul Bull UN 1982 66 70 12264850

[B30] Hakulinen T Hansluwka H Lopez AD Nakada T Global and Regional Mortality Patterns by Cause of Death in 1980 Int J Epidemiol 1986 15 226 233 10.1093/ije/15.2.226 3721685

[B31] Hull T Lopez A Rohde J A framework for estimating causes of death in Indonesia [causes of death in Indonesia] Majalah Demografi Indones 1981 8 77 125 12265996

[B32] Bulatao RA Stephens PW Global estimates and projections of mortality by cause, 1970-2015. The World Bank 1992

[B33] Black RE Morris SS Bryce J Where and why are 10 million children dying every year? The Lancet 2003 361 2226 2234 10.1016/S0140-6736(03)13779-8 12842379

[B34] Murray C Lopez A The global burden of disease: a comprehensive assessment of mortality and disability from diseases, injuries, and risk factors in 1990 and projected in 2020 1996 Cambridge, MA: Harvard Univ. Press

[B35] Wilmoth J Mathers C Sayc L Millsd S Maternal deaths drop by one-third from 1990 to 2008: a United Nations analysis Bull World Health Organ 2010 88 718 718A 10.2471/BLT.10.082446 20931050 PMC2947050

[B36] Hogan MC Foreman KJ Naghavi M Ahn SY Wang M Makela SM Lopez AD Lozano R Murray CJ Maternal mortality for 181 countries, 1980-2008: a systematic analysis of progress towards Millennium Development Goal 5 The Lancet 2010 375 1609 1623 10.1016/S0140-6736(10)60518-1 20382417

[B37] Boschi-Pinto C Lanata CF Black RE Ehiri J The Global Burden of Childhood Diarrhea Maternal and Child Health 2009 Boston, MA: Springer US 225 243

[B38] Horton R Maternal mortality: surprise, hope, and urgent action The Lancet 2010 375 1581 1582 10.1016/S0140-6736(10)60547-8 20382418

[B39] Graham WJ Braunholtz DA Campbell OM New modelled estimates of maternal mortality The Lancet 2010 375 1963 10.1016/S0140-6736(10)60918-X 20569833

[B40] AbouZahr C New estimates of maternal mortality and how to interpret them: choice or confusion? Reproductive Health Matters 2011 19 117 128 10.1016/S0968-8080(11)37550-7 21555092

[B41] Byass P The Imperfect World of Global Health Estimates PLoS Med 2010 7 e1001006 10.1371/journal.pmed.1001006 21152416 PMC2994666

[B42] Rajaratnam JK Marcus JR Flaxman AD Wang H Levin-Rector A Dwyer L Costa M Lopez AD Murray CJ Neonatal, postneonatal, childhood, and under-5 mortality for 187 countries, 1970-2010: a systematic analysis of progress towards Millennium Development Goal 4 The Lancet 2010 375 1988 2008 10.1016/S0140-6736(10)60703-9 20546887

[B43] Finucane MM Stevens GA Cowan MJ Danaei G Lin JK Paciorek CJ Singh GM Gutierrez HR Lu Y Bahalim AN Farzadfar F Riley LM Ezzati M National, regional, and global trends in body-mass index since 1980: systematic analysis of health examination surveys and epidemiological studies with 960 country-years and 9·1 million participants The Lancet 2011 377 557 567 10.1016/S0140-6736(10)62037-5 PMC4472365 21295846

[B44] Farzadfar F Finucane MM Danaei G Pelizzari PM Cowan MJ Paciorek CJ Singh GM Lin JK Stevens GA Riley LM Ezzati M National, regional, and global trends in serum total cholesterol since 1980: systematic analysis of health examination surveys and epidemiological studies with 321 country-years and 3·0 million participants The Lancet 2011 377 578 586 10.1016/S0140-6736(10)62038-7 21295847

[B45] Danaei G Finucane MM Lin JK Singh GM Paciorek CJ Cowan MJ Farzadfar F Stevens GA Lim SS Riley LM Ezzati M National, regional, and global trends in systolic blood pressure since 1980: systematic analysis of health examination surveys and epidemiological studies with 786 country-years and 5·4 million participants The Lancet 2011 377 568 577 10.1016/S0140-6736(10)62036-3 21295844

[B46] Janssen F Kunst AE ICD coding changes and discontinuities in trends in cause-specific mortality in six European countries, 1950-99 Bull World Health Organ 2004 82 904 913 15654404 PMC2623106

[B47] Feuer EJ Merrill RM Hankey BF Cancer Surveillance Series: Interpreting Trends in Prostate Cancer--Part II: Cause of Death Misclassification and the Recent Rise and Fall in Prostate Cancer Mortality Journal of the National Cancer Institute 1999 91 1025 1032 10.1093/jnci/91.12.1025 10379965

[B48] Horon IL Underreporting of Maternal Deaths on Death Certificates and the Magnitude of the Problem of Maternal Mortality Am J Public Health 2005 95 478 482 10.2105/AJPH.2004.040063 15727980 PMC1449205

[B49] Naghavi M Makela S Foreman K O'Brien J Pourmalek F Lozano R Algorithms for enhancing public health utility of national causes-of-death data Popul Health Metrics 2010 8 9 10.1186/1478-7954-8-9 PMC2873308 20459720

[B50] Lopez AD Project DCP Global burden of disease and risk factors 2006 World Bank Publications

[B51] Ahern RM Lozano R Naghavi M Foreman K Gakidou E Murray CJ Improving the public health utility of global cardiovascular mortality data: the rise of ischemic heart disease Popul Health Metrics 2011 9 8 10.1186/1478-7954-9-8 PMC3064613 21406100

[B52] Birnbaum J Murray C Lozano R Exposing misclassified HIV/AIDS deaths in South Africa Bulletin of the World Health Organization 2011 89 278 285 10.2471/BLT.11.086280 21479092 PMC3066530

[B53] Mathers C Bernard C Iburg K Inoue M Ma Fat D Shibuya K Stein C Tomijima N Xu H Global burden of disease in 2002: data sources, methods and results 2003 Geneva: GPE Discussion PAper- No. 54-World Health Organization

[B54] Bell RM Koren Y Lessons from the Netflix prize challenge SIGKDD Explor Newsl 2007 9 75 79 10.1145/1345448.1345465

[B55] Bell R Koren Y Volinsky C The BellKor solution to the Netflix Prize http://www.netflixprize.com/assets/GrandPrize2009_BPC_BellKor.pdf

[B56] Bell RM Koren Y Volinsky C All together now: A perspective on the NETFLIX PRIZE CHANCE 2010 23 24 24 10.1007/s00144-010-0005-2

[B57] Ajami NK Duan Q Sorooshian S An integrated hydrologic Bayesian multimodel combination framework: Confronting input, parameter, and model structural uncertainty in hydrologic prediction Water Resour Res 2007 43 19

[B58] Taylor JW Buizza R Neural network load forecasting with weather ensemble predictions IEEE Transactions on Power Systems 2002 17 626 632 10.1109/TPWRS.2002.800906

[B59] Krishnamurti TN Kishtawal CM Zhang Z LaRow T Bachiochi D Williford E Gadgil S Surendran S Multimodel Ensemble Forecasts for Weather and Seasonal Climate J Climate 2000 13 4196 4216 10.1175/1520-0442(2000)013<4196:MEFFWA>2.0.CO;2 10477515

[B60] Chen Y Yang B Abraham A Flexible neural trees ensemble for stock index modeling Neurocomputing 2007 70 697 703 10.1016/j.neucom.2006.10.005

[B61] Castillo O Melin P Simulation and forecasting complex economic time series usingneural networks and fuzzy logic International Joint Conference on Neural Networks, 2001. Proceedings. IJCNN '01 2001 3 IEEE 1805 1810

[B62] Wöhling T Vrugt JA Combining multiobjective optimization and Bayesian model averaging to calibrate forecast ensembles of soil hydraulic models Water Resour Res 2008 44 18

[B63] Gneiting T Raftery AE Strictly Proper Scoring Rules, Prediction, and Estimation Journal of the American Statistical Association 2007 102 359 378 10.1198/016214506000001437

[B64] Vrugt J Robinson BA Treatment of uncertainty using ensemble methods: Comparison of sequential data assimilation and Bayesian model averaging Water Resour Res 2007 43 W01411.1 W01411.15

[B65] Raftery A Gneiting T Balabdaoui F Polakowski M Using Bayesian model averaging to calibrate forecast ensembles Monthly Weather Review 2005 133 1155 74 10.1175/MWR2906.1

[B66] Hoeting JA Madigan D Raftery AE Volinsky CT Bayesian Model Averaging: A Tutorial Statistical Science 1999 14 382 401 10.1214/ss/1009212519

[B67] Claeskens G Hjort NL Model Selection and Model Averaging 2008 Cambridge University Press

[B68] King G "Truth" Is Stranger than Prediction, More Questionable than Causal Inference American Journal of Political Science 1991 35 1047 1053 10.2307/2111506

[B69] Power M The predictive validation of ecological and environmental models Ecological Modelling 1993 68 33 50 10.1016/0304-3800(93)90106-3

[B70] Snee RD Validation of Regression Models: Methods and Examples Technometrics 1977 19 415 428 10.2307/1267881

[B71] Dey DK Gelfand AE Swartz TB Vlachos PK A simulation-intensive approach for checking hierarchical models Test 1998 7 325 346 10.1007/BF02565116

[B72] Tashman LJ Out-of-sample tests of forecasting accuracy: an analysis and review International Journal of Forecasting 16 437 450

[B73] Fushiki T Estimation of prediction error by using K-fold cross-validation Stat Comput 2009 21 137 146

[B74] Zhang P Model Selection Via Multifold Cross Validation The Annals of Statistics 1993 21 299 313 10.1214/aos/1176349027

[B75] Shao J Linear Model Selection by Cross-Validation Journal of the American Statistical Association 1993 88 486 494 10.2307/2290328

[B76] Gompertz B On the nature of the function expressive of the law of human mortality, and on a new mode of determining the value of Life Contingencies Philosophical transaction of the Royal society of London 1825 London: W. Nicol 513 10.1098/rstb.2014.0379 PMC4360127 25750242

[B77] Derksen S Keselman H Backward, forward and stepwise automated subset selection algorithms: frequency of obtaining authentic and noise variables British journal of mathematical & statistical psychology 1992 45 265 82 10.1111/j.2044-8317.1992.tb00992.x22287099

[B78] Blanchet FG Legendre P Borcard D FORWARD SELECTION OF EXPLANATORY VARIABLES Ecology 2008 89 2623 2632 10.1890/07-0986.1 18831183

[B79] Meinshausen N High-dimensional graphs and variable selection with the Lasso Ann Statist 2006 34 1436 1462 10.1214/009053606000000281

[B80] Zou H Hastie T Regularization and variable selection via the elastic net Journal of the Royal Statistical Society: Series B (Statistical Methodology) 2005 67 301 320 10.1111/j.1467-9868.2005.00503.x

[B81] Smith M Kohn R Nonparametric regression using Bayesian variable selection Journal of Econometrics 1996 75 317 343 10.1016/0304-4076(95)01763-1

[B82] Allen DM The Relationship between Variable Selection and Data Agumentation and a Method for Prediction Technometrics 1974 16 125 127 10.2307/1267500

[B83] Greenland S Modeling and variable selection in epidemiologic analysis Am J Public Health 1989 79 340 349 10.2105/AJPH.79.3.340 2916724 PMC1349563

[B84] Daszykowski M Kaczmarek K Vander Heyden Y Walczak B Robust statistics in data analysis -- A review: Basic concepts Chemometrics and Intelligent Laboratory Systems 2007 85 203 219 10.1016/j.chemolab.2006.06.016

[B85] Rasmussen CE Bousquet O, Luxburg U, Rätsch G Berlin Gaussian Processes in Machine Learning Advanced Lectures on Machine Learning 2004 3176 Heidelberg: Springer Berlin Heidelberg 63 71 10.1007/978-3-540-28650-9_4

[B86] Rajaratnam JK Marcus JR Levin-Rector A Chalupka AN Wang H Dwyer L Costa M Lopez AD Murray CJ Worldwide mortality in men and women aged 15-59 years from 1970 to 2010: a systematic analysis The Lancet 2010 375 1704 1720 10.1016/S0140-6736(10)60517-X 20434763

[B87] Gneiting T Raftery AE Weather Forecasting with Ensemble Methods Science 2005 310 248 249 10.1126/science.1115255 16224011

[B88] Duan Q Ajami NK Gao X Sorooshian S Multi-model ensemble hydrologic prediction using Bayesian model averaging Advances in Water Resources 2007 30 1371 1386 10.1016/j.advwatres.2006.11.014

[B89] Lozano R Wang H Foreman KJ Rajaratnam JK Naghavi M Marcus JR Dwyer-Lindgren L Lofgren KT Phillips D Atkinson C Lopez AD Murray CJL Progress towards Millennium Development Goals 4 and 5 on maternal and child mortality: an updated systematic analysis Lancet 2011 378 1139 1165 10.1016/S0140-6736(11)61337-8 21937100

[B90] Forouzanfar MH Foreman KJ Delossantos AM Lozano R Lopez AD Murray CJL Naghavi M Breast and cervical cancer in 187 countries between 1980 and 2010: a systematic analysis Lancet 2011 378 1461 1484 10.1016/S0140-6736(11)61351-2 21924486

[B91] Murray CJ Lopez AD Black R Ahuja R Mohd Ali S Baqui A Dandona L Dantzer E Das V Dhingra U Dutta A Fawzi W Flaxman AD Gomez S Hernandez B Joshi R Kalter H Kumar A Kumar V Lozano R Lucero M Mehta S Neal B Ohno SL Prasad R Praveen D Premji Z Ramirez-Villalobos D Remolador H Riley I Romero M Said M Sanvictores D Sazawal S Tallo V Population Health Metrics Research Consortium gold standard verbal autopsy validation study: design, implementation, and development of analysis datasets Popul Health Metrics 2011 9 27 10.1186/1478-7954-9-27 PMC3160920 21816095

[B92] Murray CJ Lozano R Flaxman AD Vahdatpour A Lopez AD Robust metrics for assessing the performance of different verbal autopsy cause assignment methods in validation studies Popul Health Metrics 2011 9 28 10.1186/1478-7954-9-28 PMC3160921 21816106

