# Supplementary material for: Modeling causes of death: an integrated approach using CODEm
Source: Popul Health Metr. 2012 Jan 6;10:1. doi: 10.1186/1478-7954-10-1 (PMC3315398; doi:10.1186/1478-7954-10-1)
Supplement: Additional file 1 — Descriptions, rankings, and predictive validity metrics for maternal mortality component models. [file 1478-7954-10-1-S1.PDF]

Rank	Model Type	Dependent Variable	Covariates	Root Mean Squared Error			Proportion with Correct Trend			Draws
				In-Sample	Test 1	Test 2	In-Sample	Test 1	Test 2	
1	Spatiotemporal	Logit(CF)	Age-Specific Fertility Rate<br>Log Total Fertility Rate<br>Log LDI (I$ per capita)	0.369	0.620	0.635	0.737	0.664	0.664	139
2	Spatiotemporal	Logit(CF)	In-Facility Delivery (proportion)<br>Log Total Fertility Rate	0.368	0.619	0.624	0.737	0.662	0.665	119
3	Spatiotemporal	Logit(CF)	In-Facility Delivery (proportion)<br>Log Total Fertility Rate<br>Log LDI (I$ per capita)	0.368	0.621	0.627	0.737	0.664	0.667	102
4	Spatiotemporal	Logit(CF)	Age-Specific Fertility Rate<br>Skilled Birth Attendance (proportion)<br>Log Total Fertility Rate<br>Log LDI (I$ per capita)	0.368	0.619	0.628	0.738	0.662	0.664	87
4	Spatiotemporal	Logit(CF)	Skilled Birth Attendance (proportion)<br>Log Total Fertility Rate<br>Log LDI (I$ per capita)	0.368	0.621	0.629	0.738	0.664	0.667	87
6	Spatiotemporal	Logit(CF)	Age-Specific Fertility Rate<br>In-Facility Delivery (proportion)<br>Log Total Fertility Rate<br>Log LDI (I$ per capita)	0.368	0.619	0.627	0.737	0.661	0.664	64
6	Spatiotemporal	Logit(CF)	In-Facility Delivery (proportion)<br>Log Total Fertility Rate<br>Health System Access<br>Log LDI (I$ per capita)	0.368	0.621	0.628	0.737	0.664	0.667	64
8	Spatiotemporal	Logit(CF)	Log Total Fertility Rate<br>Log LDI (I$ per capita)	0.369	0.623	0.637	0.737	0.666	0.666	46
9	Spatiotemporal	Logit(CF)	Age-Specific Fertility Rate<br>In-Facility Delivery (proportion)<br>Log Total Fertility Rate<br>Health System Access<br>Log LDI (I$ per capita)	0.368	0.619	0.628	0.737	0.661	0.664	40
9	Spatiotemporal	Logit(CF)	In-Facility Delivery (proportion)<br>Log Total Fertility Rate<br>Health System Access	0.368	0.620	0.624	0.737	0.661	0.665	40
11	Spatiotemporal	Logit(CF)	Age-Specific Fertility Rate<br>In-Facility Delivery (proportion)<br>Log Total Fertility Rate	0.368	0.618	0.623	0.737	0.660	0.663	29

12	Spatiotemporal	Logit(CF)	Age-Specific Fertility Rate<br>In-Facility Delivery (proportion)<br>Log Total Fertility Rate<br>Health System Access	0.368	0.618	0.624	0.738	0.660	0.663	25
12	Spatiotemporal	Logit(CF)	Age-Specific Fertility Rate<br>Skilled Birth Attendance (proportion)<br>Log Total Fertility Rate<br>Female Education by Age<br>Health System Access<br>Log LDI (I$ per capita)	0.368	0.619	0.627	0.736	0.660	0.664	25
12	Spatiotemporal	Logit(CF)	Skilled Birth Attendance (proportion)<br>Log Total Fertility Rate	0.369	0.620	0.625	0.737	0.661	0.665	25
15	Spatiotemporal	Logit(CF)	Age-Specific Fertility Rate<br>Skilled Birth Attendance (proportion)<br>Log Total Fertility Rate	0.368	0.618	0.624	0.738	0.660	0.663	15
16	Spatiotemporal	Logit(CF)	Log Total Fertility Rate	0.369	0.623	0.636	0.736	0.662	0.664	13
17	Spatiotemporal	Logit(CF)	Age-Specific Fertility Rate<br>Log Total Fertility Rate	0.369	0.621	0.635	0.737	0.660	0.662	11
17	Spatiotemporal	Logit(CF)	Age-Specific Fertility Rate<br>Skilled Birth Attendance (proportion)<br>Log Total Fertility Rate<br>Female Education by Age<br>Log Neonatal Death Rate (per 1000)<br>Log LDI (I$ per capita)	0.368	0.617	0.627	0.735	0.658	0.662	11
19	Spatiotemporal	Logit(CF)	Age-Specific Fertility Rate<br>Skilled Birth Attendance (proportion)<br>Log Total Fertility Rate<br>Female Education by Age<br>Health System Access	0.368	0.617	0.624	0.736	0.657	0.662	8
20	Spatiotemporal	Logit(CF)	Log Total Fertility Rate<br>Antenatal Care (4 visits) Coverage (proportion)<br>HIV Prevalence, ARV-Adjusted<br>Log LDI (I$ per capita)	0.369	0.624	0.634	0.737	0.661	0.662	7
21	Spatiotemporal	Logit(CF)	Age-Specific Fertility Rate<br>Skilled Birth Attendance (proportion)<br>Log Total Fertility Rate<br>Female Education by Age<br>Log Neonatal Death Rate (per 1000)	0.368	0.616	0.626	0.735	0.655	0.660	6

22	Spatiotemporal	Logit(CF)	Age-Specific Fertility Rate<br>Skilled Birth Attendance (proportion)<br>Log Total Fertility Rate<br>HIV Prevalence, ARV-Adjusted<br>Female Education by Age<br>Log LDI (I$ per capita)	0.368	0.620	0.626	0.737	0.656	0.661	5
23	Spatiotemporal	Logit(CF)	Skilled Birth Attendance (proportion)<br>Log Total Fertility Rate<br>HIV Prevalence, ARV-Adjusted<br>Health System Access<br>Log LDI (I$ per capita)	0.369	0.623	0.627	0.737	0.658	0.663	4
24	Spatiotemporal	Logit(CF)	Age-Specific Fertility Rate<br>Log Total Fertility Rate<br>Antenatal Care (4 visits) Coverage (proportion)<br>HIV Prevalence, ARV-Adjusted<br>Log LDI (I$ per capita)	0.369	0.623	0.634	0.737	0.658	0.660	4
25	Spatiotemporal	Logit(CF)	Age-Specific Fertility Rate<br>Skilled Birth Attendance (proportion)<br>Log Total Fertility Rate<br>HIV Prevalence, ARV-Adjusted<br>Female Education by Age	0.368	0.618	0.622	0.737	0.654	0.659	3
26	Spatiotemporal	Logit(CF)	Age-Specific Fertility Rate<br>Skilled Birth Attendance (proportion)<br>Log LDI (I$ per capita)	0.368	0.631	0.638	0.738	0.660	0.663	3
26	Spatiotemporal	Logit(CF)	Age-Specific Fertility Rate<br>Skilled Birth Attendance (proportion)<br>Log Total Fertility Rate<br>HIV Prevalence, ARV-Adjusted<br>Health System Access<br>Log LDI (I$ per capita)	0.368	0.621	0.627	0.737	0.657	0.661	3
28	Spatiotemporal	Logit(CF)	Age-Specific Fertility Rate<br>Skilled Birth Attendance (proportion)<br>Log Total Fertility Rate<br>HIV Prevalence, ARV-Adjusted<br>Health System Access	0.368	0.620	0.623	0.737	0.655	0.660	2
29	Spatiotemporal	Logit(CF)	Age-Specific Fertility Rate<br>In-Facility Delivery (proportion)<br>Log LDI (I$ per capita)	0.368	0.630	0.635	0.738	0.660	0.662	2

30	Spatiotemporal	Logit(CF)	Age-Specific Fertility Rate<br>Skilled Birth Attendance (proportion)<br>Log Total Fertility Rate<br>HIV Prevalence, ARV-Adjusted<br>Log Neonatal Death Rate (per 1000)<br>Log LDI (I$ per capita)	0.368	0.619	0.626	0.735	0.652	0.658	1
30	Spatiotemporal	Logit(CF)	Log Total Fertility Rate<br>Antenatal Care (4 visits) Coverage (proportion)<br>HIV Prevalence, ARV-Adjusted	0.369	0.626	0.633	0.736	0.658	0.661	1
32	Spatiotemporal	Logit(CF)	Skilled Birth Attendance (proportion)<br>Log Total Fertility Rate<br>HIV Prevalence, ARV-Adjusted<br>Health System Access	0.369	0.622	0.623	0.737	0.656	0.661	1
33	Spatiotemporal	Logit(CF)	In-Facility Delivery (proportion)<br>Log Total Fertility Rate<br>HIV Prevalence, ARV-Adjusted<br>Female Education by Age<br>Log Neonatal Death Rate (per 1000)<br>Log LDI (I$ per capita)	0.368	0.621	0.625	0.735	0.655	0.660	1
34	Spatiotemporal	Logit(CF)	In-Facility Delivery (proportion)<br>Log Total Fertility Rate<br>HIV Prevalence, ARV-Adjusted<br>Female Education by Age<br>Log Neonatal Death Rate (per 1000)	0.368	0.619	0.623	0.735	0.652	0.658	1
35	Spatiotemporal	Logit(CF)	Age-Specific Fertility Rate<br>In-Facility Delivery (proportion)<br>Log Total Fertility Rate<br>HIV Prevalence, ARV-Adjusted<br>Female Education by Age<br>Log Neonatal Death Rate (per 1000)	0.368	0.617	0.623	0.734	0.650	0.656	1
35	Spatiotemporal	Logit(CF)	In-Facility Delivery (proportion)<br>Log LDI (I$ per capita)	0.368	0.649	0.645	0.738	0.663	0.664	1
37	Spatiotemporal	Logit(CF)	Age-Specific Fertility Rate<br>Log Total Fertility Rate<br>Antenatal Care (4 visits) Coverage (proportion)<br>HIV Prevalence, ARV-Adjusted	0.369	0.623	0.632	0.736	0.656	0.659	0

38	Spatiotemporal	Logit(CF)	Age-Specific Fertility Rate<br>Skilled Birth Attendance (proportion)<br>Log Total Fertility Rate<br>HIV Prevalence, ARV-Adjusted<br>Log Neonatal Death Rate (per 1000)	0.368	0.618	0.624	0.734	0.649	0.655	0
39	Spatiotemporal	Logit(CF)	Age-Specific Fertility Rate<br>Log Total Fertility Rate<br>HIV Prevalence, ARV-Adjusted<br>Female Education by Age<br>Health System Access<br>Log Neonatal Death Rate (per 1000)<br>Log LDI (I$ per capita)	0.368	0.620	0.629	0.734	0.652	0.658	0
40	Spatiotemporal	Logit(CF)	Skilled Birth Attendance (proportion)<br>Log Total Fertility Rate<br>HIV Prevalence, ARV-Adjusted<br>Female Education by Age<br>Log Neonatal Death Rate (per 1000)	0.368	0.620	0.625	0.735	0.651	0.657	0
41	Spatiotemporal	Logit(CF)	Log Total Fertility Rate<br>HIV Prevalence, ARV-Adjusted<br>Female Education by Age<br>Health System Access<br>Log Neonatal Death Rate (per 1000)<br>Log LDI (I$ per capita)	0.368	0.622	0.630	0.734	0.654	0.660	0
42	Spatiotemporal	Logit(CF)	Age-Specific Fertility Rate<br>Log LDI (I$ per capita)	0.368	0.638	0.653	0.738	0.659	0.661	0
43	Spatiotemporal	Logit(CF)	Skilled Birth Attendance (proportion)<br>Log LDI (I$ per capita)	0.368	0.650	0.649	0.740	0.662	0.664	0
44	Mixed Effects	Logit(CF)	Age-Specific Fertility Rate<br>In-Facility Delivery (proportion)<br>Log Total Fertility Rate<br>Log LDI (I$ per capita)	0.486	0.657	0.650	0.662	0.663	0.665	0
45	Spatiotemporal	Logit(CF)	Age-Specific Fertility Rate<br>In-Facility Delivery (proportion)	0.368	0.630	0.631	0.737	0.656	0.660	0
46	Spatiotemporal	Logit(CF)	Age-Specific Fertility Rate<br>Log Total Fertility Rate<br>HIV Prevalence, ARV-Adjusted<br>Female Education by Age<br>Health System Access<br>Log Neonatal Death Rate (per 1000)	0.368	0.620	0.628	0.733	0.648	0.655	0

46	Spatiotemporal	Logit(CF)	Skilled Birth Attendance (proportion)<br>HIV Prevalence, ARV-Adjusted<br>Female Education by Age<br>Health System Access<br>Log LDI (I$ per capita)	0.368	0.643	0.641	0.738	0.659	0.662	0
48	Mixed Effects	Logit(CF)	Age-Specific Fertility Rate<br>In-Facility Delivery (proportion)<br>Log Total Fertility Rate<br>Health System Access<br>Log LDI (I$ per capita)	0.486	0.658	0.650	0.662	0.663	0.664	0
49	Mixed Effects	Logit(CF)	Age-Specific Fertility Rate<br>Log Total Fertility Rate<br>Log LDI (I$ per capita)	0.486	0.658	0.657	0.663	0.663	0.664	0
50	Mixed Effects	Logit(CF)	Age-Specific Fertility Rate<br>Skilled Birth Attendance (proportion)<br>Log Total Fertility Rate<br>Log LDI (I$ per capita)	0.486	0.657	0.651	0.662	0.662	0.664	0
51	Spatiotemporal	Logit(CF)	Log Total Fertility Rate<br>HIV Prevalence, ARV-Adjusted<br>Female Education by Age<br>Health System Access<br>Log Neonatal Death Rate (per 1000)	0.368	0.622	0.629	0.733	0.651	0.657	0
52	Spatiotemporal	Logit(CF)	Age-Specific Fertility Rate<br>Skilled Birth Attendance (proportion)	0.368	0.632	0.634	0.738	0.655	0.660	0
52	Spatiotemporal	Logit(CF)	Age-Specific Fertility Rate<br>Skilled Birth Attendance (proportion)<br>HIV Prevalence, ARV-Adjusted<br>Female Education by Age<br>Health System Access<br>Log LDI (I$ per capita)	0.368	0.629	0.634	0.738	0.654	0.659	0
52	Mixed Effects	Logit(CF)	Age-Specific Fertility Rate<br>Skilled Birth Attendance (proportion)<br>Log Total Fertility Rate<br>Female Education by Age<br>Health System Access<br>Log LDI (I$ per capita)	0.486	0.658	0.651	0.661	0.662	0.664	0

52	Mixed Effects	Logit(CF)	Age-Specific Fertility Rate<br>Skilled Birth Attendance (proportion)<br>Log Total Fertility Rate<br>Female Education by Age<br>Log Neonatal Death Rate (per 1000)<br>Log LDI (I$ per capita)	0.486	0.657	0.651	0.662	0.662	0.664	0
52	Mixed Effects	Logit(CF)	In-Facility Delivery (proportion)<br>Log Total Fertility Rate<br>Log LDI (I$ per capita)	0.488	0.661	0.651	0.666	0.666	0.668	0
57	Spatiotemporal	Logit(CF)	Age-Specific Fertility Rate<br>In-Facility Delivery (proportion)<br>HIV Prevalence, ARV-Adjusted<br>Health System Access<br>Log LDI (I$ per capita)	0.368	0.631	0.634	0.737	0.655	0.659	0
58	Spatiotemporal	Logit(CF)	Skilled Birth Attendance (proportion)<br>Health System Access<br>Log Neonatal Death Rate (per 1000)<br>Log LDI (I$ per capita)	0.368	0.640	0.642	0.734	0.657	0.660	0
59	Mixed Effects	Logit(CF)	Age-Specific Fertility Rate<br>In-Facility Delivery (proportion)<br>Log Total Fertility Rate	0.486	0.658	0.648	0.661	0.662	0.665	0
59	Mixed Effects	Logit(CF)	In-Facility Delivery (proportion)<br>Log Total Fertility Rate<br>Health System Access<br>Log LDI (I$ per capita)	0.488	0.661	0.652	0.667	0.666	0.668	0
59	Mixed Effects	Logit(CF)	Skilled Birth Attendance (proportion)<br>Log Total Fertility Rate<br>Log LDI (I$ per capita)	0.488	0.661	0.652	0.666	0.666	0.668	0
62	Spatiotemporal	Logit(CF)	In-Facility Delivery (proportion)	0.368	0.650	0.640	0.739	0.659	0.663	0
63	Mixed Effects	Logit(CF)	In-Facility Delivery (proportion)<br>Log Total Fertility Rate	0.488	0.661	0.650	0.665	0.665	0.667	0
64	Spatiotemporal	Logit(CF)	In-Facility Delivery (proportion)<br>HIV Prevalence, ARV-Adjusted<br>Health System Access<br>Log LDI (I$ per capita)	0.368	0.647	0.641	0.737	0.658	0.661	0
65	Mixed Effects	Logit(CF)	Log Total Fertility Rate<br>Log LDI (I$ per capita)	0.488	0.662	0.659	0.667	0.667	0.668	0
65	Mixed Effects	Logit(CF)	Skilled Birth Attendance (proportion)<br>Log Total Fertility Rate	0.488	0.662	0.650	0.664	0.664	0.668	0

67	Mixed Effects	Logit(CF)	Age-Specific Fertility Rate<br>Skilled Birth Attendance (proportion)<br>Log Total Fertility Rate	0.486	0.659	0.649	0.662	0.662	0.664	0
68	Spatiotemporal	Logit(CF)	Age-Specific Fertility Rate<br>Antenatal Care (4 visits) Coverage (proportion)<br>HIV Prevalence, ARV-Adjusted<br>Log LDI (I$ per capita)	0.369	0.637	0.647	0.737	0.655	0.658	0
69	Spatiotemporal	Logit(CF)	Age-Specific Fertility Rate<br>In-Facility Delivery (proportion)<br>HIV Prevalence, ARV-Adjusted<br>Female Education by Age<br>Log Neonatal Death Rate (per 1000)	0.368	0.624	0.627	0.733	0.646	0.652	0
70	Mixed Effects	Logit(CF)	In-Facility Delivery (proportion)<br>Log Total Fertility Rate<br>Health System Access	0.488	0.662	0.650	0.665	0.665	0.667	0
71	Spatiotemporal	Logit(CF)	Age-Specific Fertility Rate<br>HIV Prevalence, ARV-Adjusted<br>Female Education by Age<br>Health System Access<br>Log Neonatal Death Rate (per 1000)<br>Log LDI (I$ per capita)	0.368	0.627	0.634	0.732	0.650	0.654	0
71	Spatiotemporal	Logit(CF)	Age-Specific Fertility Rate<br>Skilled Birth Attendance (proportion)<br>HIV Prevalence, ARV-Adjusted<br>Female Education by Age<br>Health System Access	0.368	0.628	0.629	0.736	0.650	0.656	0
73	Spatiotemporal	Logit(CF)	Age-Specific Fertility Rate<br>In-Facility Delivery (proportion)<br>HIV Prevalence, ARV-Adjusted<br>Health System Access	0.368	0.631	0.629	0.737	0.652	0.657	0
74	Mixed Effects	Logit(CF)	Age-Specific Fertility Rate<br>In-Facility Delivery (proportion)<br>Log Total Fertility Rate<br>Health System Access	0.486	0.659	0.649	0.661	0.661	0.664	0
74	Spatiotemporal	Logit(CF)	Age-Specific Fertility Rate<br>Skilled Birth Attendance (proportion)<br>HIV Prevalence, ARV-Adjusted<br>Female Education by Age<br>Log Neonatal Death Rate (per 1000)	0.368	0.624	0.629	0.733	0.646	0.651	0

74	Mixed Effects	Logit(CF)	Log Total Fertility Rate<br>Antenatal Care (4 visits) Coverage (proportion)<br>HIV Prevalence, ARV-Adjusted<br>Log LDI (I$ per capita)	0.486	0.662	0.654	0.662	0.663	0.665	0
77	Spatiotemporal	Logit(CF)	Age-Specific Fertility Rate<br>Antenatal Care (4 visits) Coverage (proportion)<br>Log Neonatal Death Rate (per 1000)	0.368	0.627	0.641	0.728	0.649	0.653	0
77	Mixed Effects	Logit(CF)	Age-Specific Fertility Rate<br>Log Total Fertility Rate<br>Antenatal Care (4 visits) Coverage (proportion)<br>HIV Prevalence, ARV-Adjusted<br>Log LDI (I$ per capita)	0.485	0.659	0.653	0.661	0.660	0.663	0
79	Mixed Effects	Logit(CF)	Age-Specific Fertility Rate<br>Skilled Birth Attendance (proportion)<br>Log Total Fertility Rate<br>Female Education by Age<br>Log Neonatal Death Rate (per 1000)	0.486	0.658	0.649	0.661	0.660	0.664	0
80	Spatiotemporal	Logit(CF)	Age-Specific Fertility Rate<br>HIV Prevalence, ARV-Adjusted<br>Female Education by Age<br>Health System Access<br>Log Neonatal Death Rate (per 1000)	0.368	0.627	0.633	0.730	0.646	0.651	0
80	Mixed Effects	Logit(CF)	Age-Specific Fertility Rate<br>Log Total Fertility Rate	0.487	0.662	0.657	0.662	0.663	0.665	0
82	Mixed Effects	Logit(CF)	Log Total Fertility Rate	0.488	0.666	0.659	0.665	0.667	0.668	0
82	Spatiotemporal	Logit(CF)	Skilled Birth Attendance (proportion)<br>HIV Prevalence, ARV-Adjusted<br>Female Education by Age<br>Health System Access	0.368	0.642	0.635	0.738	0.655	0.660	0
84	Mixed Effects	Logit(CF)	Age-Specific Fertility Rate<br>Skilled Birth Attendance (proportion)<br>Log Total Fertility Rate<br>Female Education by Age<br>Health System Access	0.486	0.659	0.649	0.661	0.661	0.665	0
85	Spatiotemporal	Logit(CF)	Skilled Birth Attendance (proportion)	0.368	0.652	0.643	0.740	0.657	0.662	0
86	Spatiotemporal	Logit(CF)	In-Facility Delivery (proportion)<br>HIV Prevalence, ARV-Adjusted<br>Female Education by Age<br>Log Neonatal Death Rate (per 1000)<br>Log LDI (I$ per capita)	0.368	0.637	0.636	0.733	0.653	0.656	0

87	Mixed Effects	Logit(CF)	Age-Specific Fertility Rate<br>Skilled Birth Attendance (proportion)<br>Log Total Fertility Rate<br>HIV Prevalence, ARV-Adjusted<br>Female Education by Age<br>Log LDI (I$ per capita)	0.485	0.657	0.648	0.659	0.659	0.662	0
88	Mixed Effects	Logit(CF)	Age-Specific Fertility Rate<br>Skilled Birth Attendance (proportion)<br>Log Total Fertility Rate<br>HIV Prevalence, ARV-Adjusted<br>Health System Access<br>Log LDI (I$ per capita)	0.485	0.657	0.647	0.658	0.658	0.662	0
88	Spatiotemporal	Ln(Rate)	In-Facility Delivery (proportion)<br>Log Total Fertility Rate<br>HIV Prevalence, ARV-Adjusted<br>Female Education by Age<br>Log LDI (I$ per capita)	0.371	0.628	0.646	0.730	0.634	0.636	0
88	Spatiotemporal	Logit(CF)	Skilled Birth Attendance (proportion)<br>HIV Prevalence, ARV-Adjusted<br>Female Education by Age<br>Log Neonatal Death Rate (per 1000)<br>Log LDI (I$ per capita)	0.368	0.638	0.638	0.733	0.652	0.656	0
88	Spatiotemporal	Logit(CF)	Skilled Birth Attendance (proportion)<br>Health System Access<br>Log Neonatal Death Rate (per 1000)	0.368	0.640	0.639	0.733	0.653	0.657	0
88	Mixed Effects	Logit(CF)	Skilled Birth Attendance (proportion)<br>Log Total Fertility Rate<br>HIV Prevalence, ARV-Adjusted<br>Health System Access<br>Log LDI (I$ per capita)	0.486	0.660	0.648	0.661	0.660	0.664	0
93	Spatiotemporal	Logit(CF)	Age-Specific Fertility Rate	0.368	0.645	0.659	0.736	0.654	0.657	0
93	Spatiotemporal	Logit(CF)	In-Facility Delivery (proportion)<br>HIV Prevalence, ARV-Adjusted<br>Health System Access	0.368	0.648	0.635	0.738	0.655	0.660	0
95	Spatiotemporal	Ln(Rate)	Age-Specific Fertility Rate<br>In-Facility Delivery (proportion)<br>Log Total Fertility Rate<br>HIV Prevalence, ARV-Adjusted<br>Female Education by Age<br>Log LDI (I$ per capita)	0.371	0.628	0.646	0.732	0.633	0.636	0

95	Mixed Effects	Logit(CF)	Age-Specific Fertility Rate<br>Skilled Birth Attendance (proportion)<br>Log Total Fertility Rate<br>HIV Prevalence, ARV-Adjusted<br>Log Neonatal Death Rate (per 1000)<br>Log LDI (I$ per capita)	0.485	0.657	0.647	0.659	0.658	0.661	0
97	Mixed Effects	Logit(CF)	In-Facility Delivery (proportion)<br>Log Total Fertility Rate<br>HIV Prevalence, ARV-Adjusted<br>Female Education by Age<br>Log Neonatal Death Rate (per 1000)<br>Log LDI (I$ per capita)	0.486	0.660	0.649	0.662	0.660	0.663	0
97	Mixed Effects	Logit(CF)	Log Total Fertility Rate<br>Antenatal Care (4 visits) Coverage (proportion)<br>HIV Prevalence, ARV-Adjusted	0.486	0.666	0.653	0.662	0.662	0.665	0
99	Spatiotemporal	Ln(Rate)	Skilled Birth Attendance (proportion)<br>Log Total Fertility Rate<br>HIV Prevalence, ARV-Adjusted<br>Female Education by Age<br>Log LDI (I$ per capita)	0.371	0.629	0.647	0.731	0.634	0.636	0
100	Spatiotemporal	Logit(CF)	Age-Specific Fertility Rate<br>Antenatal Care (4 visits) Coverage (proportion)<br>HIV Prevalence, ARV-Adjusted	0.368	0.643	0.648	0.736	0.652	0.656	0
101	Spatiotemporal	Logit(CF)	In-Facility Delivery (proportion)<br>HIV Prevalence, ARV-Adjusted<br>Female Education by Age<br>Log Neonatal Death Rate (per 1000)	0.368	0.636	0.633	0.732	0.649	0.653	0
102	Spatiotemporal	Ln(Rate)	Age-Specific Fertility Rate<br>Skilled Birth Attendance (proportion)<br>Log Total Fertility Rate<br>HIV Prevalence, ARV-Adjusted<br>Female Education by Age<br>Log LDI (I$ per capita)	0.371	0.629	0.648	0.732	0.633	0.636	0
103	Spatiotemporal	Ln(Rate)	In-Facility Delivery (proportion)<br>Log Total Fertility Rate<br>HIV Prevalence, ARV-Adjusted<br>Log Neonatal Death Rate (per 1000)<br>Log LDI (I$ per capita)	0.370	0.621	0.635	0.726	0.629	0.633	0

103	Spatiotemporal	Logit(CF)	Skilled Birth Attendance (proportion)<br>HIV Prevalence, ARV-Adjusted<br>Female Education by Age<br>Log Neonatal Death Rate (per 1000)	0.368	0.637	0.635	0.732	0.648	0.652	0
105	Mixed Effects	Logit(CF)	In-Facility Delivery (proportion)<br>Log Total Fertility Rate<br>HIV Prevalence, ARV-Adjusted<br>Female Education by Age<br>Log Neonatal Death Rate (per 1000)	0.486	0.661	0.647	0.661	0.659	0.662	0
105	Spatiotemporal	Ln(Rate)	Log Total Fertility Rate<br>Antenatal Care (4 visits) Coverage (proportion)<br>HIV Prevalence, ARV-Adjusted<br>Female Education by Age<br>Log LDI (I$ per capita)	0.371	0.635	0.659	0.729	0.635	0.637	0
107	Spatiotemporal	Ln(Rate)	Age-Specific Fertility Rate<br>In-Facility Delivery (proportion)<br>Log Total Fertility Rate<br>HIV Prevalence, ARV-Adjusted<br>Log Neonatal Death Rate (per 1000)<br>Log LDI (I$ per capita)	0.370	0.620	0.635	0.726	0.628	0.631	0
108	Mixed Effects	Logit(CF)	Skilled Birth Attendance (proportion)<br>Log Total Fertility Rate<br>HIV Prevalence, ARV-Adjusted<br>Health System Access	0.486	0.662	0.646	0.660	0.659	0.663	0
109	Mixed Effects	Logit(CF)	Age-Specific Fertility Rate<br>Skilled Birth Attendance (proportion)<br>Log Total Fertility Rate<br>HIV Prevalence, ARV-Adjusted<br>Log Neonatal Death Rate (per 1000)	0.485	0.658	0.645	0.658	0.657	0.661	0
110	Mixed Effects	Logit(CF)	Age-Specific Fertility Rate<br>In-Facility Delivery (proportion)<br>Log Total Fertility Rate<br>HIV Prevalence, ARV-Adjusted<br>Female Education by Age<br>Log Neonatal Death Rate (per 1000)	0.485	0.658	0.646	0.658	0.657	0.660	0
110	Mixed Effects	Logit(CF)	Age-Specific Fertility Rate<br>Log Total Fertility Rate<br>Antenatal Care (4 visits) Coverage (proportion)<br>HIV Prevalence, ARV-Adjusted	0.485	0.663	0.652	0.660	0.660	0.662	0

110	Mixed Effects	Logit(CF)	Skilled Birth Attendance (proportion)<br>Log Total Fertility Rate<br>HIV Prevalence, ARV-Adjusted<br>Female Education by Age<br>Log Neonatal Death Rate (per 1000)	0.486	0.661	0.647	0.660	0.659	0.663	0
113	Mixed Effects	Logit(CF)	Age-Specific Fertility Rate<br>Skilled Birth Attendance (proportion)<br>Log Total Fertility Rate<br>HIV Prevalence, ARV-Adjusted<br>Female Education by Age	0.485	0.659	0.646	0.659	0.657	0.662	0
114	Spatiotemporal	Ln(Rate)	In-Facility Delivery (proportion)<br>Skilled Birth Attendance (proportion)<br>Log Total Fertility Rate<br>HIV Prevalence, ARV-Adjusted<br>Log Neonatal Death Rate (per 1000)<br>Log LDI (I$ per capita)	0.370	0.622	0.637	0.726	0.629	0.633	0
115	Spatiotemporal	Ln(Rate)	Skilled Birth Attendance (proportion)<br>Log Total Fertility Rate<br>HIV Prevalence, ARV-Adjusted<br>Health System Access<br>Log LDI (I$ per capita)	0.371	0.633	0.649	0.731	0.633	0.634	0
116	Mixed Effects	Logit(CF)	Age-Specific Fertility Rate<br>Log Total Fertility Rate<br>HIV Prevalence, ARV-Adjusted<br>Female Education by Age<br>Health System Access<br>Log Neonatal Death Rate (per 1000)<br>Log LDI (I$ per capita)	0.485	0.659	0.650	0.658	0.657	0.661	0
117	Spatiotemporal	Ln(Rate)	Log Total Fertility Rate<br>HIV Prevalence, ARV-Adjusted<br>Female Education by Age<br>Health System Access<br>Log Neonatal Death Rate (per 1000)<br>Log LDI (I$ per capita)	0.371	0.624	0.643	0.725	0.629	0.635	0
117	Mixed Effects	Logit(CF)	Log Total Fertility Rate<br>HIV Prevalence, ARV-Adjusted<br>Female Education by Age<br>Health System Access<br>Log Neonatal Death Rate (per 1000)<br>Log LDI (I$ per capita)	0.486	0.663	0.652	0.661	0.659	0.664	0

119	Spatiotemporal	Ln(Rate)	Age-Specific Fertility Rate<br>Log Total Fertility Rate<br>Antenatal Care (4 visits) Coverage (proportion)<br>HIV Prevalence, ARV-Adjusted<br>Log LDI (I$ per capita)	0.371	0.638	0.663	0.730	0.634	0.634	0
119	Mixed Effects	Logit(CF)	Age-Specific Fertility Rate<br>Skilled Birth Attendance (proportion)<br>Log Total Fertility Rate<br>HIV Prevalence, ARV-Adjusted<br>Health System Access	0.485	0.660	0.645	0.658	0.657	0.662	0
121	Spatiotemporal	Ln(Rate)	Skilled Birth Attendance (proportion)<br>Log Total Fertility Rate<br>HIV Prevalence, ARV-Adjusted<br>Log Neonatal Death Rate (per 1000)<br>Log LDI (I$ per capita)	0.371	0.622	0.636	0.726	0.628	0.632	0
122	Spatiotemporal	Ln(Rate)	Age-Specific Fertility Rate<br>Skilled Birth Attendance (proportion)<br>Log Total Fertility Rate<br>HIV Prevalence, ARV-Adjusted<br>Log Neonatal Death Rate (per 1000)<br>Log LDI (I$ per capita)	0.370	0.621	0.637	0.726	0.628	0.631	0
123	Spatiotemporal	Ln(Rate)	Age-Specific Fertility Rate<br>Skilled Birth Attendance (proportion)<br>Log Total Fertility Rate<br>HIV Prevalence, ARV-Adjusted<br>Health System Access<br>Log LDI (I$ per capita)	0.371	0.632	0.650	0.732	0.632	0.634	0
124	Spatiotemporal	Ln(Rate)	In-Facility Delivery (proportion)<br>Log Total Fertility Rate<br>HIV Prevalence, ARV-Adjusted<br>Female Education by Age	0.371	0.629	0.644	0.731	0.630	0.633	0
125	Spatiotemporal	Ln(Rate)	Age-Specific Fertility Rate<br>In-Facility Delivery (proportion)<br>Log Total Fertility Rate<br>HIV Prevalence, ARV-Adjusted<br>Female Education by Age	0.371	0.628	0.644	0.731	0.629	0.631	0

126	Spatiotemporal	Ln(Rate)	Age-Specific Fertility Rate<br>In-Facility Delivery (proportion)<br>Log Total Fertility Rate<br>HIV Prevalence, ARV-Adjusted<br>Log Neonatal Death Rate (per 1000)	0.371	0.620	0.634	0.724	0.625	0.627	0
127	Spatiotemporal	Ln(Rate)	Age-Specific Fertility Rate<br>Log Total Fertility Rate<br>HIV Prevalence, ARV-Adjusted<br>Female Education by Age<br>Health System Access<br>Log Neonatal Death Rate (per 1000)<br>Log LDI (I$ per capita)	0.370	0.623	0.643	0.726	0.628	0.632	0
128	Mixed Effects	Logit(CF)	Log Total Fertility Rate<br>HIV Prevalence, ARV-Adjusted<br>Female Education by Age<br>Health System Access<br>Log Neonatal Death Rate (per 1000)	0.486	0.665	0.650	0.660	0.658	0.663	0
129	Spatiotemporal	Ln(Rate)	In-Facility Delivery (proportion)<br>Log Total Fertility Rate<br>HIV Prevalence, ARV-Adjusted<br>Log Neonatal Death Rate (per 1000)	0.371	0.621	0.634	0.724	0.626	0.630	0
130	Spatiotemporal	Ln(Rate)	Age-Specific Fertility Rate<br>In-Facility Delivery (proportion)<br>Skilled Birth Attendance (proportion)<br>Log Total Fertility Rate<br>HIV Prevalence, ARV-Adjusted<br>Log Neonatal Death Rate (per 1000)	0.371	0.620	0.636	0.724	0.625	0.627	0
131	Mixed Effects	Logit(CF)	Age-Specific Fertility Rate<br>Log Total Fertility Rate<br>HIV Prevalence, ARV-Adjusted<br>Female Education by Age<br>Health System Access<br>Log Neonatal Death Rate (per 1000)	0.485	0.662	0.648	0.657	0.656	0.661	0
131	Spatiotemporal	Ln(Rate)	Age-Specific Fertility Rate<br>Skilled Birth Attendance (proportion)<br>Log Total Fertility Rate<br>HIV Prevalence, ARV-Adjusted<br>Log Neonatal Death Rate (per 1000)	0.370	0.620	0.635	0.723	0.625	0.627	0

133	Spatiotemporal	Ln(Rate)	In-Facility Delivery (proportion)<br>Skilled Birth Attendance (proportion)<br>Log Total Fertility Rate<br>HIV Prevalence, ARV-Adjusted<br>Log Neonatal Death Rate (per 1000)	0.371	0.622	0.636	0.724	0.626	0.629	0
133	Spatiotemporal	Ln(Rate)	Skilled Birth Attendance (proportion)<br>Log Total Fertility Rate<br>HIV Prevalence, ARV-Adjusted<br>Female Education by Age	0.371	0.630	0.645	0.731	0.629	0.631	0
135	Spatiotemporal	Ln(Rate)	Age-Specific Fertility Rate<br>In-Facility Delivery (proportion)<br>HIV Prevalence, ARV-Adjusted<br>Female Education by Age<br>Log LDI (I$ per capita)	0.371	0.642	0.657	0.732	0.631	0.634	0
135	Spatiotemporal	Ln(Rate)	Skilled Birth Attendance (proportion)<br>Log Total Fertility Rate<br>HIV Prevalence, ARV-Adjusted<br>Log Neonatal Death Rate (per 1000)	0.371	0.622	0.635	0.724	0.625	0.628	0
137	Mixed Effects	Logit(CF)	Age-Specific Fertility Rate<br>In-Facility Delivery (proportion)<br>Log LDI (I$ per capita)	0.496	0.672	0.660	0.658	0.658	0.662	0
138	Spatiotemporal	Ln(Rate)	Age-Specific Fertility Rate<br>Skilled Birth Attendance (proportion)<br>HIV Prevalence, ARV-Adjusted<br>Female Education by Age<br>Log LDI (I$ per capita)	0.371	0.644	0.660	0.732	0.630	0.634	0
139	Spatiotemporal	Ln(Rate)	Age-Specific Fertility Rate<br>Skilled Birth Attendance (proportion)<br>Log Total Fertility Rate<br>HIV Prevalence, ARV-Adjusted<br>Female Education by Age	0.371	0.629	0.646	0.732	0.628	0.630	0
140	Spatiotemporal	Ln(Rate)	Age-Specific Fertility Rate<br>Antenatal Care (4 visits) Coverage (proportion)<br>HIV Prevalence, ARV-Adjusted<br>Female Education by Age<br>Log LDI (I$ per capita)	0.371	0.650	0.674	0.731	0.632	0.635	0
141	Mixed Effects	Logit(CF)	Age-Specific Fertility Rate<br>Skilled Birth Attendance (proportion)<br>Log LDI (I$ per capita)	0.496	0.673	0.662	0.658	0.658	0.662	0

142	Mixed Effects	Logit(CF)	Age-Specific Fertility Rate<br>In-Facility Delivery (proportion)	0.496	0.674	0.659	0.659	0.658	0.662	0
143	Spatiotemporal	Ln(Rate)	Log Total Fertility Rate<br>Antenatal Care (4 visits) Coverage (proportion)<br>HIV Prevalence, ARV-Adjusted<br>Female Education by Age	0.371	0.639	0.661	0.728	0.628	0.631	0
144	Spatiotemporal	Ln(Rate)	Age-Specific Fertility Rate<br>Skilled Birth Attendance (proportion)<br>HIV Prevalence, ARV-Adjusted<br>Health System Access<br>Log LDI (I$ per capita)	0.371	0.649	0.663	0.732	0.630	0.631	0
144	Spatiotemporal	Ln(Rate)	Age-Specific Fertility Rate<br>Skilled Birth Attendance (proportion)<br>Log Total Fertility Rate<br>HIV Prevalence, ARV-Adjusted<br>Health System Access	0.371	0.634	0.648	0.731	0.627	0.628	0
144	Spatiotemporal	Ln(Rate)	Log Total Fertility Rate<br>HIV Prevalence, ARV-Adjusted<br>Female Education by Age<br>Health System Access<br>Log Neonatal Death Rate (per 1000)	0.371	0.626	0.644	0.723	0.624	0.630	0
147	Spatiotemporal	Ln(Rate)	Age-Specific Fertility Rate<br>In-Facility Delivery (proportion)<br>HIV Prevalence, ARV-Adjusted<br>Female Education by Age	0.371	0.643	0.655	0.731	0.628	0.632	0
147	Spatiotemporal	Ln(Rate)	Skilled Birth Attendance (proportion)<br>Log Total Fertility Rate<br>HIV Prevalence, ARV-Adjusted<br>Health System Access	0.371	0.635	0.647	0.731	0.627	0.628	0
149	Mixed Effects	Logit(CF)	Age-Specific Fertility Rate<br>Antenatal Care (4 visits) Coverage (proportion)<br>Log Neonatal Death Rate (per 1000)	0.494	0.675	0.665	0.659	0.658	0.661	0
150	Spatiotemporal	Ln(Rate)	Age-Specific Fertility Rate<br>In-Facility Delivery (proportion)<br>HIV Prevalence, ARV-Adjusted<br>Health System Access<br>Log LDI (I$ per capita)	0.371	0.649	0.662	0.733	0.629	0.631	0

151	Mixed Effects	Ln(Rate)	Age-Specific Fertility Rate<br>In-Facility Delivery (proportion)<br>Log Total Fertility Rate<br>HIV Prevalence, ARV-Adjusted<br>Log Neonatal Death Rate (per 1000)<br>Log LDI (I$ per capita)	0.491	0.660	0.664	0.633	0.634	0.638	0
152	Spatiotemporal	Ln(Rate)	Age-Specific Fertility Rate<br>Log Total Fertility Rate<br>HIV Prevalence, ARV-Adjusted<br>Female Education by Age<br>Health System Access<br>Log Neonatal Death Rate (per 1000)	0.370	0.625	0.643	0.722	0.623	0.628	0
152	Mixed Effects	Logit(CF)	In-Facility Delivery (proportion)<br>Log LDI (I$ per capita)	0.509	0.704	0.675	0.664	0.662	0.667	0
154	Mixed Effects	Logit(CF)	Age-Specific Fertility Rate<br>In-Facility Delivery (proportion)<br>HIV Prevalence, ARV-Adjusted<br>Female Education by Age<br>Log Neonatal Death Rate (per 1000)	0.492	0.667	0.651	0.655	0.652	0.657	0
155	Mixed Effects	Logit(CF)	Age-Specific Fertility Rate<br>Log LDI (I$ per capita)	0.500	0.681	0.682	0.657	0.658	0.659	0
156	Mixed Effects	Logit(CF)	Skilled Birth Attendance (proportion)<br>Health System Access<br>Log Neonatal Death Rate (per 1000)	0.500	0.702	0.671	0.664	0.662	0.667	0
157	Mixed Effects	Logit(CF)	In-Facility Delivery (proportion)	0.509	0.704	0.675	0.664	0.662	0.666	0
158	Spatiotemporal	Ln(Rate)	Age-Specific Fertility Rate<br>Skilled Birth Attendance (proportion)<br>HIV Prevalence, ARV-Adjusted<br>Female Education by Age	0.371	0.646	0.658	0.732	0.628	0.631	0
158	Spatiotemporal	Ln(Rate)	Skilled Birth Attendance (proportion)<br>HIV Prevalence, ARV-Adjusted<br>Female Education by Age<br>Log LDI (I$ per capita)	0.371	0.659	0.669	0.731	0.632	0.636	0
160	Mixed Effects	Logit(CF)	Age-Specific Fertility Rate<br>Skilled Birth Attendance (proportion)	0.496	0.676	0.661	0.657	0.657	0.661	0
161	Mixed Effects	Logit(CF)	Age-Specific Fertility Rate<br>Skilled Birth Attendance (proportion)<br>HIV Prevalence, ARV-Adjusted<br>Female Education by Age<br>Log Neonatal Death Rate (per 1000)	0.492	0.668	0.652	0.653	0.651	0.657	0

161	Mixed Effects	Ln(Rate)	Age-Specific Fertility Rate<br>Skilled Birth Attendance (proportion)<br>Log Total Fertility Rate<br>HIV Prevalence, ARV-Adjusted<br>Log Neonatal Death Rate (per 1000)<br>Log LDI (I$ per capita)	0.491	0.661	0.664	0.632	0.633	0.637	0
163	Spatiotemporal	Ln(Rate)	Age-Specific Fertility Rate<br>In-Facility Delivery (proportion)<br>HIV Prevalence, ARV-Adjusted<br>Log Neonatal Death Rate (per 1000)<br>Log LDI (I$ per capita)	0.371	0.629	0.641	0.723	0.622	0.627	0
164	Spatiotemporal	Ln(Rate)	Age-Specific Fertility Rate<br>HIV Prevalence, ARV-Adjusted<br>Female Education by Age<br>Health System Access<br>Log Neonatal Death Rate (per 1000)<br>Log LDI (I$ per capita)	0.371	0.632	0.650	0.723	0.624	0.629	0
164	Spatiotemporal	Ln(Rate)	Age-Specific Fertility Rate<br>In-Facility Delivery (proportion)<br>HIV Prevalence, ARV-Adjusted<br>Log Neonatal Death Rate (per 1000)	0.371	0.629	0.639	0.721	0.621	0.624	0
164	Mixed Effects	Ln(Rate)	In-Facility Delivery (proportion)<br>Log Total Fertility Rate<br>HIV Prevalence, ARV-Adjusted<br>Log Neonatal Death Rate (per 1000)<br>Log LDI (I$ per capita)	0.492	0.662	0.663	0.633	0.633	0.638	0
167	Mixed Effects	Logit(CF)	Age-Specific Fertility Rate<br>HIV Prevalence, ARV-Adjusted<br>Female Education by Age<br>Health System Access<br>Log Neonatal Death Rate (per 1000)<br>Log LDI (I$ per capita)	0.490	0.672	0.655	0.655	0.652	0.658	0
168	Spatiotemporal	Ln(Rate)	Age-Specific Fertility Rate<br>Skilled Birth Attendance (proportion)<br>HIV Prevalence, ARV-Adjusted<br>Log Neonatal Death Rate (per 1000)<br>Log LDI (I$ per capita)	0.371	0.630	0.643	0.723	0.622	0.626	0

169	Mixed Effects	Logit(CF)	Age-Specific Fertility Rate<br>Antenatal Care (4 visits) Coverage (proportion)<br>HIV Prevalence, ARV-Adjusted<br>Log LDI (I$ per capita)	0.492	0.681	0.666	0.657	0.656	0.661	0
170	Spatiotemporal	Ln(Rate)	In-Facility Delivery (proportion)<br>HIV Prevalence, ARV-Adjusted<br>Female Education by Age<br>Log Neonatal Death Rate (per 1000)<br>Log LDI (I$ per capita)	0.370	0.641	0.648	0.724	0.625	0.632	0
171	Spatiotemporal	Ln(Rate)	Age-Specific Fertility Rate<br>Skilled Birth Attendance (proportion)<br>HIV Prevalence, ARV-Adjusted<br>Log Neonatal Death Rate (per 1000)	0.371	0.630	0.641	0.721	0.620	0.624	0
172	Mixed Effects	Logit(CF)	Skilled Birth Attendance (proportion)<br>Health System Access<br>Log Neonatal Death Rate (per 1000)<br>Log LDI (I$ per capita)	0.500	0.705	0.672	0.664	0.660	0.665	0
173	Mixed Effects	Logit(CF)	Age-Specific Fertility Rate	0.502	0.691	0.688	0.656	0.657	0.659	0
173	Spatiotemporal	Ln(Rate)	Age-Specific Fertility Rate<br>Antenatal Care (4 visits) Coverage (proportion)<br>HIV Prevalence, ARV-Adjusted<br>Log Neonatal Death Rate (per 1000)<br>Log LDI (I$ per capita)	0.371	0.635	0.654	0.720	0.623	0.626	0
173	Mixed Effects	Logit(CF)	Skilled Birth Attendance (proportion)<br>Log LDI (I$ per capita)	0.510	0.705	0.678	0.662	0.660	0.664	0
176	Mixed Effects	Logit(CF)	Age-Specific Fertility Rate<br>In-Facility Delivery (proportion)<br>HIV Prevalence, ARV-Adjusted<br>Health System Access<br>Log LDI (I$ per capita)	0.490	0.673	0.653	0.655	0.651	0.658	0
176	Mixed Effects	Logit(CF)	Age-Specific Fertility Rate<br>Skilled Birth Attendance (proportion)<br>HIV Prevalence, ARV-Adjusted<br>Female Education by Age<br>Health System Access<br>Log LDI (I$ per capita)	0.490	0.672	0.653	0.654	0.651	0.658	0

176	Mixed Effects	Ln(Rate)	In-Facility Delivery (proportion)<br>Skilled Birth Attendance (proportion)<br>Log Total Fertility Rate<br>HIV Prevalence, ARV-Adjusted<br>Log Neonatal Death Rate (per 1000)<br>Log LDI (I$ per capita)	0.492	0.663	0.665	0.633	0.633	0.637	0
179	Mixed Effects	Ln(Rate)	Skilled Birth Attendance (proportion)<br>Log Total Fertility Rate<br>HIV Prevalence, ARV-Adjusted<br>Log Neonatal Death Rate (per 1000)<br>Log LDI (I$ per capita)	0.492	0.662	0.664	0.631	0.632	0.637	0
180	Mixed Effects	Logit(CF)	Age-Specific Fertility Rate<br>HIV Prevalence, ARV-Adjusted<br>Female Education by Age<br>Health System Access<br>Log Neonatal Death Rate (per 1000)	0.490	0.674	0.654	0.655	0.651	0.658	0
180	Spatiotemporal	Ln(Rate)	Age-Specific Fertility Rate<br>Log Total Fertility Rate<br>Antenatal Care (4 visits) Coverage (proportion)<br>HIV Prevalence, ARV-Adjusted	0.371	0.645	0.669	0.729	0.625	0.626	0
180	Mixed Effects	Logit(CF)	In-Facility Delivery (proportion)<br>HIV Prevalence, ARV-Adjusted<br>Female Education by Age<br>Log Neonatal Death Rate (per 1000)	0.500	0.687	0.661	0.657	0.655	0.659	0
183	Spatiotemporal	Ln(Rate)	Age-Specific Fertility Rate<br>HIV Prevalence, ARV-Adjusted<br>Female Education by Age<br>Health System Access<br>Log Neonatal Death Rate (per 1000)	0.371	0.634	0.650	0.719	0.620	0.625	0
184	Spatiotemporal	Ln(Rate)	Skilled Birth Attendance (proportion)<br>HIV Prevalence, ARV-Adjusted<br>Female Education by Age	0.371	0.662	0.666	0.730	0.632	0.633	0
185	Mixed Effects	Ln(Rate)	Age-Specific Fertility Rate<br>Log Total Fertility Rate<br>HIV Prevalence, ARV-Adjusted<br>Female Education by Age<br>Health System Access<br>Log Neonatal Death Rate (per 1000)<br>Log LDI (I$ per capita)	0.491	0.664	0.671	0.632	0.632	0.636	0

186	Mixed Effects	Logit(CF)	Age-Specific Fertility Rate<br>Skilled Birth Attendance (proportion)<br>HIV Prevalence, ARV-Adjusted<br>Female Education by Age<br>Health System Access	0.490	0.674	0.652	0.654	0.650	0.657	0
187	Spatiotemporal	Ln(Rate)	In-Facility Delivery (proportion)<br>Skilled Birth Attendance (proportion)<br>HIV Prevalence, ARV-Adjusted<br>Log Neonatal Death Rate (per 1000)<br>Log LDI (I$ per capita)	0.371	0.644	0.649	0.723	0.624	0.629	0
188	Mixed Effects	Ln(Rate)	In-Facility Delivery (proportion)<br>Log Total Fertility Rate<br>HIV Prevalence, ARV-Adjusted<br>Female Education by Age<br>Log LDI (I$ per capita)	0.494	0.672	0.675	0.633	0.635	0.638	0
189	Spatiotemporal	Ln(Rate)	In-Facility Delivery (proportion)<br>HIV Prevalence, ARV-Adjusted<br>Female Education by Age<br>Log Neonatal Death Rate (per 1000)	0.371	0.642	0.645	0.721	0.623	0.629	0
189	Mixed Effects	Ln(Rate)	In-Facility Delivery (proportion)<br>Log Total Fertility Rate<br>HIV Prevalence, ARV-Adjusted<br>Log Neonatal Death Rate (per 1000)	0.492	0.664	0.661	0.631	0.632	0.635	0
189	Mixed Effects	Ln(Rate)	In-Facility Delivery (proportion)<br>Skilled Birth Attendance (proportion)<br>Log Total Fertility Rate<br>HIV Prevalence, ARV-Adjusted<br>Log Neonatal Death Rate (per 1000)	0.492	0.665	0.663	0.631	0.632	0.635	0
192	Spatiotemporal	Ln(Rate)	Age-Specific Fertility Rate<br>Antenatal Care (4 visits) Coverage (proportion)<br>HIV Prevalence, ARV-Adjusted<br>Log Neonatal Death Rate (per 1000)	0.371	0.637	0.656	0.715	0.617	0.621	0
192	Spatiotemporal	Ln(Rate)	Age-Specific Fertility Rate<br>In-Facility Delivery (proportion)<br>HIV Prevalence, ARV-Adjusted<br>Health System Access	0.371	0.653	0.660	0.732	0.626	0.627	0
192	Mixed Effects	Logit(CF)	Age-Specific Fertility Rate<br>In-Facility Delivery (proportion)<br>HIV Prevalence, ARV-Adjusted<br>Health System Access	0.490	0.675	0.652	0.655	0.651	0.658	0

195	Mixed Effects	Ln(Rate)	Age-Specific Fertility Rate<br>In-Facility Delivery (proportion)<br>Log Total Fertility Rate<br>HIV Prevalence, ARV-Adjusted<br>Log Neonatal Death Rate (per 1000)	0.492	0.662	0.661	0.629	0.630	0.634	0
195	Spatiotemporal	Ln(Rate)	Age-Specific Fertility Rate<br>Skilled Birth Attendance (proportion)<br>HIV Prevalence, ARV-Adjusted<br>Health System Access	0.371	0.654	0.662	0.733	0.626	0.628	0
195	Spatiotemporal	Ln(Rate)	Skilled Birth Attendance (proportion)<br>HIV Prevalence, ARV-Adjusted<br>Log Neonatal Death Rate (per 1000)<br>Log LDI (I$ per capita)	0.371	0.644	0.650	0.723	0.624	0.627	0
198	Mixed Effects	Logit(CF)	Age-Specific Fertility Rate<br>Antenatal Care (4 visits) Coverage (proportion)<br>HIV Prevalence, ARV-Adjusted	0.492	0.690	0.668	0.657	0.655	0.660	0
198	Mixed Effects	Ln(Rate)	Age-Specific Fertility Rate<br>In-Facility Delivery (proportion)<br>Log Total Fertility Rate<br>HIV Prevalence, ARV-Adjusted<br>Female Education by Age<br>Log LDI (I$ per capita)	0.494	0.671	0.675	0.633	0.634	0.637	0
200	Spatiotemporal	Ln(Rate)	Age-Specific Fertility Rate<br>In-Facility Delivery (proportion)<br>Skilled Birth Attendance (proportion)<br>HIV Prevalence, ARV-Adjusted<br>Health System Access	0.371	0.654	0.663	0.732	0.626	0.627	0
201	Mixed Effects	Ln(Rate)	Skilled Birth Attendance (proportion)<br>Log Total Fertility Rate<br>HIV Prevalence, ARV-Adjusted<br>Female Education by Age<br>Log LDI (I$ per capita)	0.494	0.673	0.676	0.632	0.634	0.638	0
201	Mixed Effects	Ln(Rate)	Skilled Birth Attendance (proportion)<br>Log Total Fertility Rate<br>HIV Prevalence, ARV-Adjusted<br>Log Neonatal Death Rate (per 1000)	0.492	0.664	0.661	0.631	0.631	0.635	0
203	Mixed Effects	Logit(CF)	Skilled Birth Attendance (proportion)<br>HIV Prevalence, ARV-Adjusted<br>Female Education by Age<br>Log Neonatal Death Rate (per 1000)	0.499	0.688	0.663	0.655	0.653	0.659	0

204	Mixed Effects	Ln(Rate)	Age-Specific Fertility Rate<br>Skilled Birth Attendance (proportion)<br>Log Total Fertility Rate<br>HIV Prevalence, ARV-Adjusted<br>Female Education by Age<br>Log LDI (I$ per capita)	0.494	0.672	0.677	0.632	0.633	0.636	0
204	Mixed Effects	Logit(CF)	In-Facility Delivery (proportion)<br>HIV Prevalence, ARV-Adjusted<br>Female Education by Age<br>Log Neonatal Death Rate (per 1000)<br>Log LDI (I$ per capita)	0.499	0.689	0.661	0.656	0.654	0.659	0
204	Mixed Effects	Logit(CF)	Skilled Birth Attendance (proportion)	0.510	0.706	0.678	0.660	0.659	0.664	0
207	Mixed Effects	Ln(Rate)	Age-Specific Fertility Rate<br>In-Facility Delivery (proportion)<br>Skilled Birth Attendance (proportion)<br>Log Total Fertility Rate<br>HIV Prevalence, ARV-Adjusted<br>Log Neonatal Death Rate (per 1000)	0.492	0.663	0.663	0.629	0.630	0.634	0
207	Mixed Effects	Ln(Rate)	Log Total Fertility Rate<br>HIV Prevalence, ARV-Adjusted<br>Female Education by Age<br>Health System Access<br>Log Neonatal Death Rate (per 1000)<br>Log LDI (I$ per capita)	0.492	0.666	0.671	0.633	0.632	0.637	0
209	Spatiotemporal	Ln(Rate)	In-Facility Delivery (proportion)<br>Skilled Birth Attendance (proportion)<br>HIV Prevalence, ARV-Adjusted<br>Log Neonatal Death Rate (per 1000)	0.371	0.645	0.647	0.719	0.623	0.626	0
210	Spatiotemporal	Ln(Rate)	Skilled Birth Attendance (proportion)<br>HIV Prevalence, ARV-Adjusted<br>Log Neonatal Death Rate (per 1000)	0.371	0.645	0.647	0.719	0.623	0.627	0
211	Mixed Effects	Ln(Rate)	Age-Specific Fertility Rate<br>Skilled Birth Attendance (proportion)<br>Log Total Fertility Rate<br>HIV Prevalence, ARV-Adjusted<br>Log Neonatal Death Rate (per 1000)	0.492	0.663	0.661	0.629	0.630	0.634	0
212	Spatiotemporal	Ln(Rate)	In-Facility Delivery (proportion)<br>HIV Prevalence, ARV-Adjusted<br>Health System Access<br>Log LDI (I$ per capita)	0.370	0.668	0.671	0.731	0.631	0.632	0

213	Mixed Effects	Logit(CF)	Skilled Birth Attendance (proportion)<br>HIV Prevalence, ARV-Adjusted<br>Female Education by Age<br>Health System Access	0.496	0.694	0.663	0.658	0.654	0.661	0
214	Spatiotemporal	Ln(Rate)	Age-Specific Fertility Rate<br>Antenatal Care (4 visits) Coverage (proportion)<br>HIV Prevalence, ARV-Adjusted<br>Female Education by Age	0.371	0.659	0.678	0.729	0.627	0.631	0
215	Spatiotemporal	Ln(Rate)	Skilled Birth Attendance (proportion)<br>HIV Prevalence, ARV-Adjusted<br>Health System Access<br>Log LDI (I$ per capita)	0.370	0.668	0.673	0.732	0.631	0.631	0
216	Mixed Effects	Logit(CF)	Skilled Birth Attendance (proportion)<br>HIV Prevalence, ARV-Adjusted<br>Female Education by Age<br>Log Neonatal Death Rate (per 1000)<br>Log LDI (I$ per capita)	0.499	0.689	0.663	0.655	0.652	0.658	0
217	Mixed Effects	Ln(Rate)	Log Total Fertility Rate<br>Antenatal Care (4 visits) Coverage (proportion)<br>HIV Prevalence, ARV-Adjusted<br>Female Education by Age<br>Log LDI (I$ per capita)	0.493	0.679	0.684	0.638	0.639	0.640	0
217	Mixed Effects	Ln(Rate)	Skilled Birth Attendance (proportion)<br>Log Total Fertility Rate<br>HIV Prevalence, ARV-Adjusted<br>Health System Access<br>Log LDI (I$ per capita)	0.493	0.674	0.675	0.632	0.633	0.636	0
219	Mixed Effects	Ln(Rate)	Age-Specific Fertility Rate<br>Log Total Fertility Rate<br>Antenatal Care (4 visits) Coverage (proportion)<br>HIV Prevalence, ARV-Adjusted<br>Log LDI (I$ per capita)	0.493	0.679	0.686	0.636	0.639	0.639	0
219	Mixed Effects	Logit(CF)	In-Facility Delivery (proportion)<br>HIV Prevalence, ARV-Adjusted<br>Health System Access	0.496	0.697	0.664	0.658	0.654	0.661	0

221	Mixed Effects	Ln(Rate)	Age-Specific Fertility Rate<br>Skilled Birth Attendance (proportion)<br>Log Total Fertility Rate<br>HIV Prevalence, ARV-Adjusted<br>Health System Access<br>Log LDI (I$ per capita)	0.493	0.673	0.675	0.632	0.632	0.635	0
222	Spatiotemporal	Ln(Rate)	In-Facility Delivery (proportion)<br>Skilled Birth Attendance (proportion)<br>HIV Prevalence, ARV-Adjusted<br>Health System Access<br>Log LDI (I$ per capita)	0.370	0.669	0.673	0.732	0.630	0.631	0
223	Mixed Effects	Logit(CF)	Skilled Birth Attendance (proportion)<br>HIV Prevalence, ARV-Adjusted<br>Female Education by Age<br>Health System Access<br>Log LDI (I$ per capita)	0.496	0.696	0.664	0.657	0.653	0.660	0
224	Mixed Effects	Logit(CF)	In-Facility Delivery (proportion)<br>HIV Prevalence, ARV-Adjusted<br>Health System Access<br>Log LDI (I$ per capita)	0.496	0.699	0.664	0.657	0.654	0.660	0
225	Spatiotemporal	Ln(Rate)	In-Facility Delivery (proportion)<br>Log Total Fertility Rate<br>Log LDI (I$ per capita)	0.371	0.651	0.660	0.731	0.608	0.615	0
226	Spatiotemporal	Ln(Rate)	Skilled Birth Attendance (proportion)<br>Log Total Fertility Rate<br>Log LDI (I$ per capita)	0.371	0.652	0.661	0.732	0.609	0.614	0
227	Spatiotemporal	Ln(Rate)	Age-Specific Fertility Rate<br>In-Facility Delivery (proportion)<br>Log Total Fertility Rate<br>Log LDI (I$ per capita)	0.371	0.651	0.661	0.732	0.607	0.615	0
228	Spatiotemporal	Ln(Rate)	Age-Specific Fertility Rate<br>Skilled Birth Attendance (proportion)<br>Log Total Fertility Rate<br>Log LDI (I$ per capita)	0.371	0.652	0.662	0.733	0.607	0.614	0
229	Spatiotemporal	Ln(Rate)	In-Facility Delivery (proportion)<br>Skilled Birth Attendance (proportion)<br>Log Total Fertility Rate<br>Log LDI (I$ per capita)	0.371	0.653	0.662	0.731	0.608	0.614	0

230	Spatiotemporal	Ln(Rate)	Age-Specific Fertility Rate<br>In-Facility Delivery (proportion)<br>Skilled Birth Attendance (proportion)<br>Log Total Fertility Rate<br>Log LDI (I$ per capita)	0.371	0.653	0.663	0.732	0.607	0.614	0
231	Mixed Effects	Ln(Rate)	Age-Specific Fertility Rate<br>In-Facility Delivery (proportion)<br>Log Total Fertility Rate<br>HIV Prevalence, ARV-Adjusted<br>Female Education by Age	0.496	0.673	0.670	0.630	0.630	0.633	0
232	Spatiotemporal	Ln(Rate)	Age-Specific Fertility Rate<br>In-Facility Delivery (proportion)<br>Skilled Birth Attendance (proportion)<br>Log Neonatal Death Rate (per 1000)<br>Log LDI (I$ per capita)	0.371	0.645	0.652	0.722	0.600	0.609	0
233	Mixed Effects	Ln(Rate)	In-Facility Delivery (proportion)<br>Log Total Fertility Rate<br>HIV Prevalence, ARV-Adjusted<br>Female Education by Age	0.496	0.675	0.670	0.629	0.630	0.633	0
234	Mixed Effects	Ln(Rate)	Age-Specific Fertility Rate<br>Skilled Birth Attendance (proportion)<br>Log Total Fertility Rate<br>HIV Prevalence, ARV-Adjusted<br>Female Education by Age	0.496	0.675	0.672	0.628	0.629	0.632	0
235	Spatiotemporal	Ln(Rate)	Age-Specific Fertility Rate<br>In-Facility Delivery (proportion)<br>Skilled Birth Attendance (proportion)<br>Log Neonatal Death Rate (per 1000)	0.371	0.646	0.650	0.719	0.598	0.606	0
235	Spatiotemporal	Ln(Rate)	In-Facility Delivery (proportion)<br>Log Total Fertility Rate	0.371	0.654	0.658	0.731	0.603	0.608	0
235	Spatiotemporal	Ln(Rate)	Skilled Birth Attendance (proportion)<br>Log Total Fertility Rate	0.371	0.655	0.660	0.731	0.604	0.608	0
238	Spatiotemporal	Ln(Rate)	In-Facility Delivery (proportion)<br>Skilled Birth Attendance (proportion)<br>Log Total Fertility Rate	0.371	0.656	0.661	0.731	0.604	0.608	0
238	Mixed Effects	Ln(Rate)	Skilled Birth Attendance (proportion)<br>Log Total Fertility Rate<br>HIV Prevalence, ARV-Adjusted<br>Female Education by Age	0.496	0.676	0.671	0.629	0.629	0.633	0

240	Spatiotemporal	Ln(Rate)	In-Facility Delivery (proportion)<br>Skilled Birth Attendance (proportion)<br>Log Total Fertility Rate<br>Female Education by Age	0.371	0.654	0.660	0.730	0.603	0.610	0
240	Mixed Effects	Ln(Rate)	Log Total Fertility Rate<br>HIV Prevalence, ARV-Adjusted<br>Female Education by Age<br>Health System Access<br>Log Neonatal Death Rate (per 1000)	0.492	0.670	0.671	0.628	0.628	0.634	0
242	Mixed Effects	Ln(Rate)	Age-Specific Fertility Rate<br>Log Total Fertility Rate<br>HIV Prevalence, ARV-Adjusted<br>Female Education by Age<br>Health System Access<br>Log Neonatal Death Rate (per 1000)	0.492	0.668	0.670	0.627	0.627	0.632	0
243	Spatiotemporal	Ln(Rate)	Age-Specific Fertility Rate<br>Skilled Birth Attendance (proportion)<br>Log Total Fertility Rate	0.371	0.655	0.660	0.732	0.603	0.608	0
244	Spatiotemporal	Ln(Rate)	Age-Specific Fertility Rate<br>In-Facility Delivery (proportion)<br>Skilled Birth Attendance (proportion)<br>Log Total Fertility Rate	0.371	0.655	0.661	0.731	0.603	0.608	0
245	Spatiotemporal	Ln(Rate)	Age-Specific Fertility Rate<br>In-Facility Delivery (proportion)<br>Log Total Fertility Rate	0.371	0.654	0.659	0.731	0.602	0.608	0
245	Spatiotemporal	Ln(Rate)	In-Facility Delivery (proportion)<br>Skilled Birth Attendance (proportion)<br>Female Education by Age<br>Log Neonatal Death Rate (per 1000)	0.371	0.657	0.654	0.718	0.603	0.610	0
247	Mixed Effects	Ln(Rate)	Age-Specific Fertility Rate<br>In-Facility Delivery (proportion)<br>HIV Prevalence, ARV-Adjusted<br>Female Education by Age<br>Log LDI (I$ per capita)	0.502	0.686	0.686	0.631	0.630	0.635	0
248	Spatiotemporal	Ln(Rate)	In-Facility Delivery (proportion)<br>HIV Prevalence, ARV-Adjusted<br>Health System Access	0.371	0.674	0.668	0.731	0.628	0.631	0

249	Mixed Effects	Ln(Rate)	Log Total Fertility Rate<br>Antenatal Care (4 visits) Coverage (proportion)<br>HIV Prevalence, ARV-Adjusted<br>Female Education by Age	0.496	0.690	0.686	0.629	0.631	0.632	0
250	Mixed Effects	Ln(Rate)	Age-Specific Fertility Rate<br>Skilled Birth Attendance (proportion)<br>HIV Prevalence, ARV-Adjusted<br>Female Education by Age<br>Log LDI (I$ per capita)	0.502	0.688	0.688	0.630	0.630	0.635	0
251	Spatiotemporal	Ln(Rate)	Age-Specific Fertility Rate<br>Log Total Fertility Rate<br>Log LDI (I$ per capita)	0.371	0.659	0.678	0.730	0.605	0.609	0
252	Mixed Effects	Ln(Rate)	Age-Specific Fertility Rate<br>Antenatal Care (4 visits) Coverage (proportion)<br>HIV Prevalence, ARV-Adjusted<br>Log Neonatal Death Rate (per 1000)<br>Log LDI (I$ per capita)	0.497	0.683	0.684	0.628	0.629	0.632	0
253	Mixed Effects	Ln(Rate)	Age-Specific Fertility Rate<br>Skilled Birth Attendance (proportion)<br>Log Total Fertility Rate<br>HIV Prevalence, ARV-Adjusted<br>Health System Access	0.494	0.680	0.674	0.628	0.628	0.631	0
254	Mixed Effects	Ln(Rate)	Skilled Birth Attendance (proportion)<br>Log Total Fertility Rate<br>HIV Prevalence, ARV-Adjusted<br>Health System Access	0.495	0.681	0.673	0.626	0.629	0.630	0
255	Mixed Effects	Ln(Rate)	Age-Specific Fertility Rate<br>In-Facility Delivery (proportion)<br>HIV Prevalence, ARV-Adjusted<br>Log Neonatal Death Rate (per 1000)<br>Log LDI (I$ per capita)	0.499	0.672	0.669	0.624	0.625	0.630	0
255	Spatiotemporal	Ln(Rate)	Skilled Birth Attendance (proportion)<br>HIV Prevalence, ARV-Adjusted<br>Health System Access	0.371	0.674	0.670	0.732	0.627	0.629	0
257	Spatiotemporal	Ln(Rate)	Log Total Fertility Rate<br>Log LDI (I$ per capita)	0.371	0.660	0.678	0.731	0.603	0.608	0

258	Mixed Effects	Ln(Rate)	Age-Specific Fertility Rate<br>Antenatal Care (4 visits) Coverage (proportion)<br>HIV Prevalence, ARV-Adjusted<br>Female Education by Age<br>Log LDI (I$ per capita)	0.500	0.698	0.698	0.632	0.631	0.635	0
258	Mixed Effects	Ln(Rate)	Age-Specific Fertility Rate<br>HIV Prevalence, ARV-Adjusted<br>Female Education by Age<br>Health System Access<br>Log Neonatal Death Rate (per 1000)<br>Log LDI (I$ per capita)	0.497	0.676	0.677	0.629	0.628	0.635	0
260	Spatiotemporal	Ln(Rate)	In-Facility Delivery (proportion)<br>Skilled Birth Attendance (proportion)<br>HIV Prevalence, ARV-Adjusted<br>Health System Access	0.371	0.676	0.670	0.732	0.627	0.630	0
261	Mixed Effects	Ln(Rate)	Skilled Birth Attendance (proportion)<br>HIV Prevalence, ARV-Adjusted<br>Female Education by Age<br>Log LDI (I$ per capita)	0.509	0.709	0.699	0.635	0.633	0.639	0
262	Mixed Effects	Ln(Rate)	In-Facility Delivery (proportion)<br>HIV Prevalence, ARV-Adjusted<br>Female Education by Age<br>Log Neonatal Death Rate (per 1000)<br>Log LDI (I$ per capita)	0.505	0.693	0.679	0.631	0.629	0.637	0
263	Mixed Effects	Ln(Rate)	Age-Specific Fertility Rate<br>Log Total Fertility Rate<br>Antenatal Care (4 visits) Coverage (proportion)<br>HIV Prevalence, ARV-Adjusted	0.495	0.695	0.693	0.627	0.629	0.629	0
263	Mixed Effects	Ln(Rate)	Age-Specific Fertility Rate<br>Skilled Birth Attendance (proportion)<br>HIV Prevalence, ARV-Adjusted<br>Log Neonatal Death Rate (per 1000)<br>Log LDI (I$ per capita)	0.499	0.673	0.671	0.623	0.624	0.629	0
265	Mixed Effects	Ln(Rate)	In-Facility Delivery (proportion)<br>HIV Prevalence, ARV-Adjusted<br>Female Education by Age<br>Log Neonatal Death Rate (per 1000)	0.505	0.694	0.679	0.631	0.629	0.637	0

266	Mixed Effects	Ln(Rate)	Age-Specific Fertility Rate<br>In-Facility Delivery (proportion)<br>HIV Prevalence, ARV-Adjusted<br>Female Education by Age	0.503	0.692	0.685	0.628	0.628	0.634	0
267	Mixed Effects	Ln(Rate)	Age-Specific Fertility Rate<br>HIV Prevalence, ARV-Adjusted<br>Female Education by Age<br>Health System Access<br>Log Neonatal Death Rate (per 1000)	0.497	0.682	0.679	0.626	0.626	0.633	0
268	Mixed Effects	Ln(Rate)	Age-Specific Fertility Rate<br>In-Facility Delivery (proportion)<br>HIV Prevalence, ARV-Adjusted<br>Log Neonatal Death Rate (per 1000)	0.499	0.675	0.668	0.623	0.625	0.629	0
268	Mixed Effects	Ln(Rate)	Skilled Birth Attendance (proportion)<br>HIV Prevalence, ARV-Adjusted<br>Female Education by Age	0.509	0.716	0.700	0.633	0.632	0.638	0
270	Mixed Effects	Ln(Rate)	Age-Specific Fertility Rate<br>In-Facility Delivery (proportion)<br>HIV Prevalence, ARV-Adjusted<br>Health System Access<br>Log LDI (I$ per capita)	0.499	0.694	0.688	0.629	0.628	0.633	0
271	Spatiotemporal	Ln(Rate)	Age-Specific Fertility Rate<br>In-Facility Delivery (proportion)<br>Log LDI (I$ per capita)	0.371	0.667	0.673	0.734	0.605	0.615	0
272	Spatiotemporal	Ln(Rate)	Age-Specific Fertility Rate<br>In-Facility Delivery (proportion)<br>Skilled Birth Attendance (proportion)<br>Log LDI (I$ per capita)	0.371	0.668	0.676	0.734	0.605	0.614	0
272	Mixed Effects	Ln(Rate)	Age-Specific Fertility Rate<br>Skilled Birth Attendance (proportion)<br>HIV Prevalence, ARV-Adjusted<br>Health System Access<br>Log LDI (I$ per capita)	0.499	0.694	0.689	0.629	0.628	0.633	0
274	Spatiotemporal	Ln(Rate)	Age-Specific Fertility Rate<br>Skilled Birth Attendance (proportion)<br>Log LDI (I$ per capita)	0.371	0.669	0.676	0.735	0.606	0.614	0
275	Mixed Effects	Ln(Rate)	Age-Specific Fertility Rate<br>Skilled Birth Attendance (proportion)<br>HIV Prevalence, ARV-Adjusted<br>Female Education by Age	0.503	0.695	0.688	0.628	0.627	0.633	0

275	Mixed Effects	Ln(Rate)	Age-Specific Fertility Rate<br>Skilled Birth Attendance (proportion)<br>HIV Prevalence, ARV-Adjusted<br>Log Neonatal Death Rate (per 1000)	0.499	0.676	0.670	0.623	0.623	0.628	0
277	Mixed Effects	Ln(Rate)	Age-Specific Fertility Rate<br>Antenatal Care (4 visits) Coverage (proportion)<br>HIV Prevalence, ARV-Adjusted<br>Log Neonatal Death Rate (per 1000)	0.497	0.690	0.687	0.624	0.624	0.629	0
278	Mixed Effects	Ln(Rate)	Age-Specific Fertility Rate<br>Antenatal Care (4 visits) Coverage (proportion)<br>HIV Prevalence, ARV-Adjusted<br>Female Education by Age	0.501	0.713	0.705	0.629	0.629	0.635	0
279	Spatiotemporal	Ln(Rate)	In-Facility Delivery (proportion)<br>Log LDI (I$ per capita)	0.371	0.684	0.682	0.733	0.608	0.614	0
280	Mixed Effects	Ln(Rate)	In-Facility Delivery (proportion)<br>Skilled Birth Attendance (proportion)<br>HIV Prevalence, ARV-Adjusted<br>Health System Access<br>Log LDI (I$ per capita)	0.504	0.718	0.702	0.629	0.628	0.634	0
281	Mixed Effects	Ln(Rate)	In-Facility Delivery (proportion)<br>HIV Prevalence, ARV-Adjusted<br>Health System Access<br>Log LDI (I$ per capita)	0.504	0.717	0.700	0.629	0.628	0.635	0
281	Mixed Effects	Ln(Rate)	In-Facility Delivery (proportion)<br>Skilled Birth Attendance (proportion)<br>HIV Prevalence, ARV-Adjusted<br>Log Neonatal Death Rate (per 1000)<br>Log LDI (I$ per capita)	0.507	0.696	0.680	0.625	0.624	0.629	0
281	Spatiotemporal	Ln(Rate)	In-Facility Delivery (proportion)<br>Skilled Birth Attendance (proportion)<br>Log LDI (I$ per capita)	0.371	0.685	0.684	0.734	0.608	0.614	0
284	Spatiotemporal	Ln(Rate)	Age-Specific Fertility Rate<br>In-Facility Delivery (proportion)	0.371	0.674	0.673	0.734	0.602	0.607	0
285	Spatiotemporal	Ln(Rate)	Skilled Birth Attendance (proportion)<br>Log LDI (I$ per capita)	0.371	0.686	0.685	0.733	0.606	0.612	0
286	Spatiotemporal	Ln(Rate)	Log Total Fertility Rate	0.371	0.669	0.687	0.726	0.593	0.602	0
287	Spatiotemporal	Ln(Rate)	Age-Specific Fertility Rate<br>Log Total Fertility Rate	0.371	0.669	0.687	0.728	0.592	0.601	0

288	Spatiotemporal	Ln(Rate)	Age-Specific Fertility Rate<br>In-Facility Delivery (proportion)<br>Skilled Birth Attendance (proportion)	0.371	0.675	0.676	0.733	0.602	0.607	0
288	Mixed Effects	Ln(Rate)	Skilled Birth Attendance (proportion)<br>HIV Prevalence, ARV-Adjusted<br>Health System Access<br>Log LDI (I$ per capita)	0.504	0.717	0.701	0.629	0.627	0.634	0
288	Mixed Effects	Ln(Rate)	Skilled Birth Attendance (proportion)<br>HIV Prevalence, ARV-Adjusted<br>Log Neonatal Death Rate (per 1000)<br>Log LDI (I$ per capita)	0.507	0.695	0.680	0.623	0.623	0.628	0
291	Mixed Effects	Ln(Rate)	Age-Specific Fertility Rate<br>In-Facility Delivery (proportion)<br>HIV Prevalence, ARV-Adjusted<br>Health System Access	0.500	0.704	0.691	0.626	0.625	0.631	0
291	Mixed Effects	Ln(Rate)	In-Facility Delivery (proportion)<br>Skilled Birth Attendance (proportion)<br>HIV Prevalence, ARV-Adjusted<br>Log Neonatal Death Rate (per 1000)	0.507	0.697	0.680	0.626	0.624	0.630	0
293	Spatiotemporal	Ln(Rate)	Age-Specific Fertility Rate<br>Skilled Birth Attendance (proportion)	0.371	0.676	0.677	0.735	0.602	0.607	0
294	Mixed Effects	Ln(Rate)	Skilled Birth Attendance (proportion)<br>HIV Prevalence, ARV-Adjusted<br>Log Neonatal Death Rate (per 1000)	0.507	0.697	0.680	0.623	0.623	0.629	0
295	Mixed Effects	Ln(Rate)	Age-Specific Fertility Rate<br>In-Facility Delivery (proportion)<br>Skilled Birth Attendance (proportion)<br>HIV Prevalence, ARV-Adjusted<br>Health System Access	0.500	0.705	0.693	0.626	0.624	0.631	0
296	Mixed Effects	Ln(Rate)	Age-Specific Fertility Rate<br>Skilled Birth Attendance (proportion)<br>HIV Prevalence, ARV-Adjusted<br>Health System Access	0.500	0.704	0.691	0.625	0.624	0.630	0
296	Spatiotemporal	Ln(Rate)	In-Facility Delivery (proportion)	0.371	0.693	0.681	0.733	0.605	0.610	0
298	Spatiotemporal	Ln(Rate)	In-Facility Delivery (proportion)<br>Skilled Birth Attendance (proportion)	0.371	0.694	0.683	0.734	0.604	0.608	0
299	Mixed Effects	Ln(Rate)	In-Facility Delivery (proportion)<br>Skilled Birth Attendance (proportion)<br>HIV Prevalence, ARV-Adjusted<br>Health System Access	0.504	0.725	0.705	0.628	0.626	0.632	0

300	Mixed Effects	Ln(Rate)	In-Facility Delivery (proportion)<br>HIV Prevalence, ARV-Adjusted<br>Health System Access	0.504	0.725	0.702	0.628	0.625	0.632	0
301	Spatiotemporal	Ln(Rate)	Age-Specific Fertility Rate<br>Log LDI (I$ per capita)	0.371	0.686	0.706	0.734	0.600	0.606	0
302	Mixed Effects	Ln(Rate)	Skilled Birth Attendance (proportion)<br>HIV Prevalence, ARV-Adjusted<br>Health System Access	0.504	0.725	0.703	0.628	0.625	0.632	0
303	Spatiotemporal	Ln(Rate)	Skilled Birth Attendance (proportion)	0.371	0.695	0.685	0.734	0.603	0.607	0
304	Mixed Effects	Ln(Rate)	Age-Specific Fertility Rate<br>In-Facility Delivery (proportion)<br>Log Total Fertility Rate<br>Log LDI (I$ per capita)	0.511	0.699	0.690	0.595	0.598	0.606	0
305	Mixed Effects	Ln(Rate)	In-Facility Delivery (proportion)<br>Log Total Fertility Rate<br>Log LDI (I$ per capita)	0.511	0.698	0.689	0.595	0.598	0.605	0
306	Mixed Effects	Ln(Rate)	Age-Specific Fertility Rate<br>In-Facility Delivery (proportion)<br>Skilled Birth Attendance (proportion)<br>Log Neonatal Death Rate (per 1000)<br>Log LDI (I$ per capita)	0.512	0.693	0.679	0.592	0.590	0.604	0
307	Mixed Effects	Ln(Rate)	Age-Specific Fertility Rate<br>In-Facility Delivery (proportion)<br>Skilled Birth Attendance (proportion)<br>Log Total Fertility Rate<br>Log LDI (I$ per capita)	0.510	0.700	0.692	0.596	0.598	0.607	0
308	Mixed Effects	Ln(Rate)	In-Facility Delivery (proportion)<br>Skilled Birth Attendance (proportion)<br>Log Total Fertility Rate<br>Log LDI (I$ per capita)	0.510	0.699	0.691	0.596	0.597	0.606	0
309	Mixed Effects	Ln(Rate)	Age-Specific Fertility Rate<br>Skilled Birth Attendance (proportion)<br>Log Total Fertility Rate<br>Log LDI (I$ per capita)	0.511	0.700	0.692	0.593	0.598	0.605	0
309	Mixed Effects	Ln(Rate)	In-Facility Delivery (proportion)<br>Log LDI (I$ per capita)	0.527	0.739	0.720	0.605	0.606	0.615	0
309	Mixed Effects	Ln(Rate)	Skilled Birth Attendance (proportion)<br>Log Total Fertility Rate<br>Log LDI (I$ per capita)	0.511	0.700	0.691	0.593	0.597	0.605	0

312	Mixed Effects	Ln(Rate)	In-Facility Delivery (proportion)<br>Skilled Birth Attendance (proportion)<br>Log LDI (I$ per capita)	0.527	0.741	0.722	0.606	0.606	0.615	0
313	Mixed Effects	Ln(Rate)	Age-Specific Fertility Rate<br>In-Facility Delivery (proportion)<br>Skilled Birth Attendance (proportion)<br>Log Neonatal Death Rate (per 1000)	0.512	0.697	0.679	0.591	0.590	0.604	0
314	Mixed Effects	Ln(Rate)	In-Facility Delivery (proportion)<br>Log Total Fertility Rate	0.513	0.704	0.686	0.588	0.592	0.600	0
315	Mixed Effects	Ln(Rate)	Age-Specific Fertility Rate<br>In-Facility Delivery (proportion)<br>Log Total Fertility Rate	0.513	0.705	0.687	0.588	0.593	0.601	0
316	Mixed Effects	Ln(Rate)	In-Facility Delivery (proportion)<br>Skilled Birth Attendance (proportion)	0.529	0.751	0.724	0.603	0.604	0.610	0
317	Mixed Effects	Ln(Rate)	Age-Specific Fertility Rate<br>Log Total Fertility Rate<br>Log LDI (I$ per capita)	0.513	0.711	0.711	0.597	0.598	0.607	0
318	Mixed Effects	Ln(Rate)	Age-Specific Fertility Rate<br>In-Facility Delivery (proportion)<br>Log LDI (I$ per capita)	0.522	0.721	0.709	0.598	0.599	0.610	0
318	Mixed Effects	Ln(Rate)	Age-Specific Fertility Rate<br>In-Facility Delivery (proportion)<br>Skilled Birth Attendance (proportion)<br>Log LDI (I$ per capita)	0.521	0.722	0.710	0.598	0.599	0.610	0
318	Mixed Effects	Ln(Rate)	Log Total Fertility Rate<br>Log LDI (I$ per capita)	0.513	0.711	0.710	0.597	0.598	0.606	0
318	Mixed Effects	Ln(Rate)	Skilled Birth Attendance (proportion)	0.532	0.753	0.725	0.603	0.603	0.610	0
322	Mixed Effects	Ln(Rate)	Age-Specific Fertility Rate<br>In-Facility Delivery (proportion)<br>Skilled Birth Attendance (proportion)<br>Log Total Fertility Rate	0.513	0.706	0.689	0.588	0.593	0.602	0
323	Mixed Effects	Ln(Rate)	In-Facility Delivery (proportion)<br>Skilled Birth Attendance (proportion)<br>Log Total Fertility Rate	0.513	0.705	0.688	0.587	0.592	0.601	0
324	Mixed Effects	Ln(Rate)	Age-Specific Fertility Rate<br>Skilled Birth Attendance (proportion)<br>Log LDI (I$ per capita)	0.523	0.724	0.713	0.597	0.599	0.610	0
324	Mixed Effects	Ln(Rate)	In-Facility Delivery (proportion)	0.529	0.750	0.721	0.602	0.602	0.609	0

324	Mixed Effects	Ln(Rate)	In-Facility Delivery (proportion)<br>Skilled Birth Attendance (proportion)<br>Female Education by Age<br>Log Neonatal Death Rate (per 1000)	0.516	0.710	0.687	0.598	0.595	0.607	0
327	Mixed Effects	Ln(Rate)	Skilled Birth Attendance (proportion)<br>Log Total Fertility Rate	0.514	0.705	0.688	0.590	0.591	0.601	0
328	Mixed Effects	Ln(Rate)	Age-Specific Fertility Rate<br>Skilled Birth Attendance (proportion)<br>Log Total Fertility Rate	0.514	0.706	0.689	0.591	0.592	0.601	0
329	Mixed Effects	Ln(Rate)	Age-Specific Fertility Rate<br>In-Facility Delivery (proportion)	0.524	0.731	0.709	0.597	0.598	0.609	0
330	Mixed Effects	Ln(Rate)	Skilled Birth Attendance (proportion)<br>Log LDI (I$ per capita)	0.529	0.742	0.724	0.599	0.600	0.608	0
331	Mixed Effects	Ln(Rate)	In-Facility Delivery (proportion)<br>Skilled Birth Attendance (proportion)<br>Log Total Fertility Rate<br>Female Education by Age	0.513	0.706	0.688	0.589	0.591	0.601	0
332	Mixed Effects	Ln(Rate)	Age-Specific Fertility Rate<br>Skilled Birth Attendance (proportion)	0.527	0.734	0.713	0.596	0.598	0.608	0
333	Spatiotemporal	Ln(Rate)	Age-Specific Fertility Rate	0.371	0.711	0.732	0.733	0.580	0.589	0
333	Mixed Effects	Ln(Rate)	Age-Specific Fertility Rate<br>In-Facility Delivery (proportion)<br>Skilled Birth Attendance (proportion)	0.524	0.732	0.711	0.596	0.598	0.609	0
335	Mixed Effects	Ln(Rate)	Log Total Fertility Rate	0.521	0.720	0.718	0.582	0.580	0.597	0
336	Mixed Effects	Ln(Rate)	Age-Specific Fertility Rate<br>Log LDI (I$ per capita)	0.534	0.750	0.752	0.593	0.593	0.603	0
336	Mixed Effects	Ln(Rate)	Age-Specific Fertility Rate<br>Log Total Fertility Rate	0.521	0.721	0.718	0.580	0.579	0.595	0
338	Mixed Effects	Ln(Rate)	Age-Specific Fertility Rate	0.550	0.774	0.779	0.569	0.572	0.584	0
